# Pore-Opening Dynamics of Single Nanometer Biovesicles at an Electrified Interface

**DOI:** 10.1021/acsnano.2c03929

**Published:** 2022-06-01

**Authors:** Xinwei Zhang, Andrew G. Ewing

**Affiliations:** Department of Chemistry and Molecular Biology, University of Gothenburg, SE-412 96 Gothenburg, Sweden

**Keywords:** biovesicular release heterogeneity, biomembrane electroporation, nanobiovesicles, vesicle impact electrochemical cytometry, numerical simulation

## Abstract

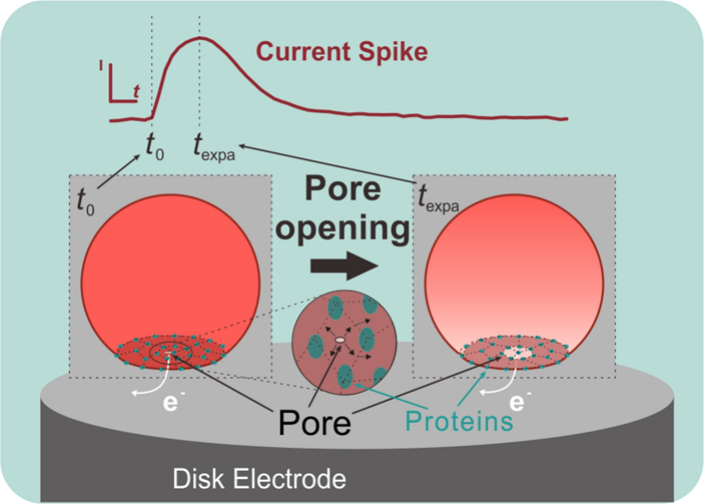

Release from nanobiovesicles via a pore generated by membrane electroporation at an electrified interface can be monitored by vesicle impact electrochemical cytometry (VIEC) and provides rich information about the various vesicular content transfer processes, including content homeostasis, intraphase content transfer, or the transient fusion of vesicles. These processes are primarily influenced by the vesicular pore-opening dynamics at the electrified interface which has not been disclosed at the single nanobiovesicle level yet. In this work, after simultaneously measuring the size and release dynamics of individual vesicles, we employed a moving mesh-finite element simulation algorithm to reconstruct the accurate pore-opening dynamics of individual vesicles with different sizes during VIEC. We investigated the expansion times and maximal pore sizes as two characteristics of different vesicles. The pore expansion times between nanobiovesicles and pure lipid liposomes were compared, and that of the nanobiovesicles is much longer than that for the liposomes, 2.1 ms vs 0.18 ms, respectively, which reflects the membrane proteins limiting the electroporation process. For the vesicles with different sizes, a positive relationship of pore size (*R*_p,max_) with the vesicle size (*R*_ves_) and also their ratio (*R*_p,max_/*R*_ves_) versus the vesicle sizes is observed. The mechanism of the pore size determination is discussed and related to the membrane proteins and the vesicle size. This work accurately describes the dynamic pore-opening process of individual vesicles which discloses the heterogeneity in electroporation of different sized vesicles. This should allow us to examine the more complicated vesicular content transfer process between intravesicular compartments.

Nanometer biovesicles are essential organelles involved in storage and transport of various cellular substances, including proteins, enzymes, hormones, neurotransmitters, and nucleic acids and also have been developed for novel systems of drug delivery.^1^ The inward and outward transfer of the chemical content of vesicles (the term “vesicles” in this paper means biovesicles, to be distinguished from the artificial vesicles, called “liposomes” in this paper), i.e., loading and release, are fundamental processes in intercellular communication, including neuronal signaling, hormonal regulation, etc. Electrochemical approaches, owing to their high time resolution, sensitivity, and strong quantitative ability, have been widely applied to investigate the transient nature of vesicular content transfer, especially in exocytosis.^2−6^ Among them, an electrochemical approach, based on the impact electrochemistry,^7−9^ for precise quantification and monitoring of the total transmitter content of individual vesicles, called vesicle impact electrochemical cytometry (VIEC), was developed and applied to various vesicles (from chromaffin,^10^ PC12,^11^ beta cells,^12^ BON cells,^13^ and fly neurons^14^), phagolysosomes,^15,16^ platelets,^17^ and liposomes.^18,19^

VIEC mimics exocytosis, but the vesicle opening is by electroporation of the vesicle membrane, which triggers all chemical contents to diffuse out from the vesicle onto the adjacent electrode. The electrode generates a high local electric field resulting in electroporation and is also used to quantitatively count molecules released in real time by measuring the electrooxidation signal in the form of a current transient or “spike”. The spike-shaped signals also provide multidimensional dynamic information about the content transfer, such as the content homeostasis,^15^ the intraphase content transfer (“membrane-halo”^20^ or “dense core-halo”^21^), or the transient fusion of vesicles.^22,23^ However, the recording of this dynamic information is primarily regulated by the pore generated during VIEC, which makes the mechanism of various intravesicular processes difficult to ascertain. This means that clarification of the pore-opening dynamics becomes a precondition for further measurement of the above complex content transfer processes in an individual vesicle.

The reconstruction of pore-opening dynamics from the spike signal was pioneered by Amatore’s group, which introduced an analytical algorithm for the similar vesicular release during exocytosis.^24−27^ Moreover, several groups adopted simulation approaches to investigate how vesicular release was influenced by the vesicular matrix,^28^ pore positions,^29^ and the distance between the pore and electrode surface.^30^ However, by either approach, the reconstruction of precise pore-opening dynamics from the current spike for individual vesicles was only partially obtained. This is because, for this reconstruction, it is necessary to know the size of each individual vesicle, but the simultaneous measurement of vesicular release dynamics and vesicle size is extremely difficult. Therefore, most work to date assumes that the vesicle size is uniform and uses the average radius of a population of vesicles to calculate their pore-opening dynamics.^28^ This assumption ignores the heterogeneity of biovesicles and their pore-opening situation, whereas our recent work has enabled this investigation of the pore opening of individual vesicles with the resistive pulse (RP) and carbon nanopore measurements that discriminate vesicles by size and content either individually or in size ranges, respectively,^31,32^ and provides the possibility of accurately reconstructing pore opening dynamics of individual vesicles with different sizes.

In this paper, in order to study the pore-opening dynamics of individual vesicles with the data collected by the RP-VIEC,^32^ we developed an algorithm based on finite element simulation. The pore-opening dynamic characteristics of each vesicle, including the pore expansion time and pore size, were correlated to their respective vesicle size and compared to those of liposomes made from pure lipids. The results show that the pore-opening time of biovesicles is significantly longer than that for liposomes and the size ratio of pore/vesicles increase with the vesicle size, showing that membrane pore opening is vesicle size-dependent and might be regulated by the vesicular membrane proteins missing on liposomes. Additionally, the degree of vesicle maturation appears to be important in pore opening. This not only leads a deeper understanding of the pore-opening dynamics of the heterogeneous biovesicles but also provides a reference for further studies on the complex regulation of vesicular release, related physiological processes, and drug delivery system development.

## Results and Discussion

### Model of Vesicular Release via Dynamic Pore Opening during VIEC

**Figure 1:**
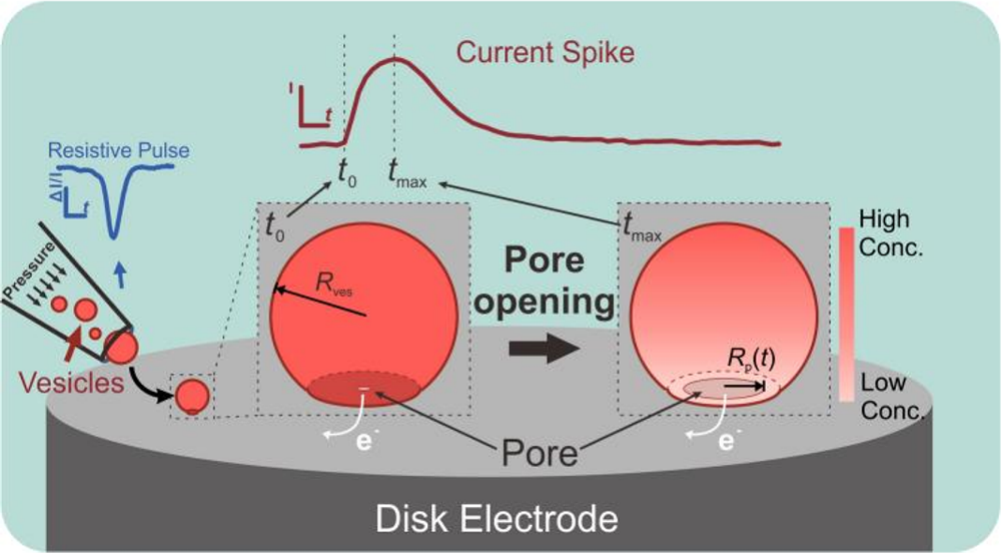
Schematic of vesicular release in VIEC following a RP measurement. This model describes the initial high concentration of catecholamine within vesicle lumen (*t* ≤ 0 ms) and its gradient formed after the pore opening (*t* > 0 ms). Here, *t*_max_ indicates time of current maxima, *R*_ves_ is the vesicle radius obtained by the RP measurement, and *R*_p_(*t*) presents the pore radius change over time. Picture is not drawn according to scale.

In VIEC, each biovesicle impacts the electrode surface and then forms a pore induced by the strong local electric field.^19,29^ The attachment is driven by an affinity of the vesicular membrane to the electrode surface, during which the affinity will induce a deformation of the vesicle and form a contact area between vesicle and electrode surface.^33,34^ Considering the strongest electric field is located at the electrode surface–vesicle interface, electroporation seems most possible within the contact region.^29,35^ However, due to the low osmolarity of the surrounding solution of vesicle (see the configuration of RP-VIEC in Methods), the osmotic pressure outward might lead to a higher membrane tension to keep the sphericity of the vesicle to some degree during the attachment and vesicular release. To assess vesicle radius, a resistive pulse (RP) measurement was performed before the VIEC. The RP-VIEC measurement and the pore-opening process are shown schematically in Figure 1.

To clarify the pore-opening dynamics during VIEC, a finite element model of the release of vesicular catecholamines via a dynamic pore and the collection of released species by a carbon fiber electrode was created referencing the model of exocytosis because of their similarity (see more details in the Methods).^24,29,30,36^ Briefly, when the pore generation starts (*t* = 0 ms), a sphere (with radius, *R*_ves_ in Figure 1 and Figure S1) is used to define the vesicle interior with the initial content, and a cylinder is placed at the bottom of the sphere to define the pore zone. The pore zone connects the vesicle interior to a narrow space between vesicle and electrode surface (see Figure S1A). At *t* > 0 ms, the pore expands over the time and its radius is defined as *R*_p_(*t*) (see Figure 1 and Figure S1B). The species concentration at the electrode surface is set as 0 M at all times to represent the diffusion-limited electrooxidation of catecholamines because the electrode potential (+700 mV vs Ag/AgCl) is set to oxidize all catecholamines reaching the surface. Owing to the concentration gradient across the pore zone, the contents within the vesicle diffuse out via the pore toward the electrode. The total content flux reaching the electrode surface can be calculated as the surface integral of diffusional flux over the pore cross-section and further converted into current according to Faraday’s law. The vesicle radius and its initial catecholamine content have been antecedently measured by the RP method and the charge of the oxidation spike by VIEC, respectively (see more experimental details in the Methods and Supporting Information),^32^ so these values are known.

Notably, this model assumes that the diffusion of contents in the vesicle are uniform including the initial concentration and diffusion coefficient. However, the dense core vesicle presents two domains (“dense core” and “halo”) which usually results in different diffusional properties, leading to a limitation of this model. As discussed previously,^32^ the experimental spikes from vesicular release events where the falling portion of the spike are better fit to a single exponential decay function are assumed to belong to nondense core vesicles. Thus, we decided to use only those spikes with a single exponential decay, classified as the nondense core vesicles, for the analysis by the finite element simulation algorithm in this work.

### An Algorithm for the Reconstruction of the Pore-Opening Dynamics

Accurate reconstruction of the pore-opening dynamics is based on the finite element model discussed above and the experimental current spike from VIEC. Generally, reconstruction is accomplished by fitting the simulated current to the experimental current spike at each time point. However, the current finite element simulations of vesicular release usually ignore all the time details of pore-opening dynamics and are simplified into fixed values after pore opening (i.e., a Heaviside function).^29,30,36^ We thus employed a moving mesh approach to facilitate the simulation of the dynamic pore opening. In contrast to the commonly used fixed mesh model, this technique can provide the time-dependent deformation of the space geometry of vesicular release, including the size of pore zone in this work (see Movie S1).

To present the pore opening, we set an interpolation function, *R*_p_(*t*), to define the pore radius change over time. The *R*_p_(*t*) is solved by estimating a series of discrete radii values (from *t* = 0 ms to the end of the spike) to compose this interpolation function which can make the simulated current fit precisely to the experimental current at each time point.

More specifically, after setting the initial condition (*t* = 0 ms) of the vesicle on the electrode surface (see Figure 1 and Figure S1A), the pore radius is set to an extremely small value (10^–10^ m in this work) to make the outflow close to 0. At the first time point (*t*_1_ = 1 × Δ*T*, Δ*T* = 0.1 ms in this work, the sampling interval of VIEC current data), the Nelder–Mead algorithm^37,38^ has been adopted to search the best-fit radius of pore (*R*_p,1_) to make the simulated current (*I*_sim,1_) closest to the experimental data. After determining the *R*_p,1_, we record this value (as the first data point of *R*_p_(*t*)), move to the next time point (*t*_2_ = *t*_1_ + Δ*T*), search the best-fit value of *R*_p,2_, and continue these steps to the end of *R*_p,n_. After obtaining all the values of *R*_p_(*t*) (*R*_p,1_ ∼ *R*_p,n_), the entire process of pore opening over time is reconstructed (see the protocol details in the Methods).

**Figure 2:**
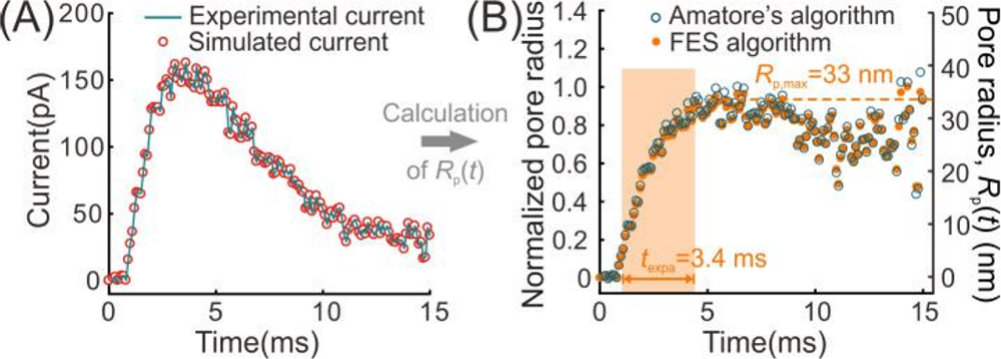
Example of the best fit *R*_p_(*t*) of an experimental spike through the finite element simulation algorithm (FESA). (A) Experimental spike (blue line) and its best fit simulated current (red circles). (B) Scatter of normalized *R*_p_(*t*) calculated by the FESA (yellow dots) and Amatore’s algorithm (blue circles). Their consistency supports the reliability of the FESA. The *R*_p_(*t*) scatter by FESA was also associated with the right-Y axis.

Some examples of experimental spikes and their fitting results are shown in Figure 2 and Figure S2. An example simulated *R*_p_(*t*) is compared to the normalized pore-opening radius obtained by Amatore’s algorithm^26^ (shown in Figure 2B), and they are almost fully consistent. However, our finite element simulation algorithm (FESA) allows calculation of the pore-opening dynamics and can be carried out with various sizes of vesicles. The flexibility in setting up the finite element model allows the algorithm to be further used to resolve some more complicated problems, such as the content transfer from the dense core to the halo within a vesicle.^36^

The FESA was used to examine the specific process of pore opening during VIEC. The calculated *R*_p_(*t*) shows that the pore keeps expanding until a plateau is reached (see Figure 2B and Figure S2). Hence, the pore-opening dynamics can be easily characterized with two key parameters, the maximum pore radius (*R*_p,max_) and the time of pore expansion (*t*_expa_) which is defined as the time of pore expanding from 5% to 95% of the *R*_p,max_. These two parameters might facilitate the analysis of the electroporation process of vesicles and further speculation of their regulation by the different vesicular membrane properties is discussed below.

### Time and Size of Pore Opening during VIEC

**Figure 3:**
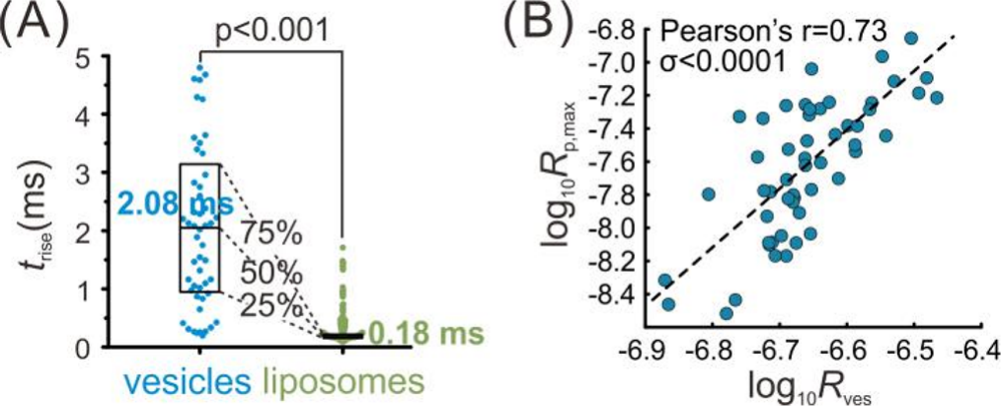
(A) Comparison between *t*_rise_ of chromaffin vesicles and liposomes during VIEC. The medians of each data set (2.08 ms for vesicles vs 0.18 ms for liposomes) are shown against their respective scatter plots. A nonparametric, two tailed Mann–Whitney test was applied to compare the two data sets, and the *p* value is included above the bar. (B) Correlation of the maxima of pore radius (*R*_p,max_) to the vesicle radius (*R*_ves_). A Pearson’s test was applied to evaluate the correlation. The expression of fitting curve is log_10_*R*_p,max_ = 3.43 × log_10_*R*_ves_ + 15.26 (unit: m).

A population of biovesicles (*n* = 53) was tested by RP-VIEC (see experimental details in the Supporting Information), and the times of current increase (*t*_rise_, 5% to 95% of the current maximum) were statistically analyzed, which is equivalent to the time of pore expansion.^25−27^ These pore expansion times are in the range of several milliseconds and long compared to previous theoretical examples of electroporation of lipid membranes where pore opening time usually ranged from approximately nanoseconds to microseconds.^39−43^ The slower temporal response of the pore opening might result from the biovesicular membrane proteins. To experimentally confirm whether the proteins affect the pore expansion rate, we compared the *t*_rise_ values of biovesicles and liposomes made from pure lipids (see experimental details in the Supporting Information) during VIEC (see Figure 3A).

Comparison of chromaffin vesicles and liposomes shows a significantly longer time for pore expansion in vesicles versus liposomes, *t*_rise_ = 2.1 ms (median, 0.9 ms as first quarter, 3.4 ms as third quarters) for vesicles (*n* = 53) *vs t*_rise_ = 0.18 ms (median, 0.16 ms as first quarter, 0.22 ms as third quarters) for liposomes (*n* = 476) . As the difference between these systems is the presence of proteins on the vesicular membranes, the results suggest that the presence of vesicular membrane proteins slow down the pore expansion.

The size of the pore formed during VIEC is another key characteristic of the pore-opening dynamics. Through the RP-VIEC measurements on chromaffin vesicles and the finite element simulation algorithm, we also estimate and plot the logarithm of maximum of the pore radius (*R*_p,max_) for each vesicle versus its respective vesicle radius (*R*_ves_) as shown in Figure 3B (see their original values in Figure S3A). The fitting results might provide a reference to estimate the pore radius of a vesicle with a certain size. The results show that *R*_p,max_ increases with the *R*_ves_, which might uncover a potential intrinsic relation between electroporation dynamics and vesicle size. To understand this relationship, a more specific discussion about the electroporation of chromaffin vesicle is required.

### Determination of Pore Size during VIEC

During VIEC, vesicles are adsorbed on the electrode surface with each deforming to generate a contact area where electroporation most possibly occurs (see Figure 1). Thus, the area of contact region might restrain the pore size and area. This contact area (*S*_c_) depends on the size and deformation degree of vesicle (expressed as *S*_c_ = *k*_d_*S*_ves_), where the *S*_ves_ is the surface area of the vesicle and equals 4π*R*_ves_^2^ and *k*_d_ is defined as a deformation coefficient to characterize the degree of deformation that is influenced by the stiffness of the vesicle membrane and the affinity force between the vesicle membrane and electrode surface.^33,34^

A correlation between the contact area (*S*_c_) and the maximal pore area (*S*_p,max_, equals π*R*_p,max_^2^) is expected. The above analysis of *t*_expa_ for vesicles strongly suggests that electroporation is at least in part regulated by membrane proteins. They might also regulate pore size. As suggested by the “membrane compartment” theory,^44^ biological membranes can be divided into small sections by membrane proteins. The proteins anchoring to the carbon fiber surface owing to various adsorption interactions^45^ might cause membrane properties similar to the those described in the model that vesicular membrane proteins anchor to the coat-proteins to form a protein complex during endocytosis and exocytosis (the tier 3 membrane section in Kusumi et al.^44^).

**Figure 4:**
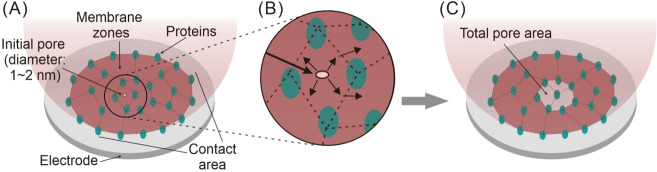
Schematic of electroporation within the contact area (dark red section). (A) Initial pore generated within a membrane section area which was formed by the vesicular membrane proteins dividing the vesicular membrane. (B) Initial pore rapidly expands, reaches the section edge, and spreads to some neighboring sections. (C) Total pore area (*S*_p,max_) consists of several membrane sections. The proteins and membrane sections are not drawn according to scale; several anchored proteins may located on a dashed line but not in a complete straight line.

After the electroporation occurs within one membrane section (see Figure 4A), the initial pore rapidly expands (approximately nanosecond to microsecond time scale as observed for electroporation within a small piece of pure lipid membrane) until the edge of the membrane section where the proteins can stabilize the pore edge is reached. Then the pore can further expand to a neighboring section of membrane after overcoming the energy barrier generated by the proteins at the section edge, and eventually more membrane sections can be transferred or included in the total pore area (see Figure 4B,C).^40,46−48^ The size of each section is difficult to estimate at present. But based on the previous work^44^ on the membrane sections formed by the protein–protein complex domains (vs the proteins-substrate complex domain in this work), the diameter appears to be from 3 to several 10s nm. This range fits our estimated pore diameter from 6 to 280 nm, considering that the final pore can contain many membrane sections.

This mechanism is consistent with the significantly slower rate of electroporation for vesicles versus liposomes. If the contact area includes a greater amount of the membrane sections, there are more sections that might be involved in pore formation and lead to a larger pore area. In this case, the total area of the pore can be expressed as follows (eq 1 is developed in the Supporting Information)
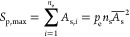
1where the *n*_e_ and *n*_s_ are the numbers of electroporated and the total small sections within the contact area, respectively; *A*_s_ is the area of individual small section and  is their average; and *p*_e_ is the irreversible electroporation probability of a small element of vesicular membrane and can be considered to be constant within the contact area of any vesicles because of the similar electric field across the similar vesicular membrane thickness even for different sized vesicles. Notably,  equals the contact area (*S*_c_) and *S*_c_ = *k*_d_*S*_ves_; thus we can deduce that  resulting in an equation for maximum pore size as eq 2.
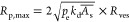
2

This formulation might provide clues to explain the positive relationship between *R*_p,max_ and *R*_ves_ and suggest the factors affecting the pore size generated on the vesicle with different sizes. The value of  (= *R*_p,max_/*R*_ves_) for each vesicle was plotted versus their respective *R*_ves_ (see Figure S3). This results in a positive relationship which appears to reflect the heterogeneity of different sized biovesicles as well.

## Conclusions

In this work, we employed a moving mesh-finite element simulation algorithm to reconstruct the accurate pore-opening dynamics of individual vesicles with different sizes during VIEC. We investigated the expansion times and maximal pore sizes, as two characteristics, of different vesicles and compared the pore expansion time between biovesicles and pure lipid liposomes. The pore expansion time of biovesicles is much longer than that for the liposomes, 2.1 ms vs 0.18 ms, respectively, which can be explained by membrane proteins slowing the electroporation process. A positive relationship of the pore sizes (*R*_p,max_) with the vesicle sizes (*R*_ves_) and also their ratio (*R*_p,max_/*R*_ves_) versus the vesicle sizes is observed. Our work accurately describes the dynamic pore-opening process of individual vesicles which discloses the heterogeneity in electroporation of different sized vesicles and the significance of RP-VIEC on single vesicle analysis. This work helps to clarify the pore expansion dynamics and provides important methodology to examine the more complicated vesicular content transfer process between intravesicular compartments.

## Methods

### Resistive Pulse-Vesicle Impact Electrochemical Cytometry (RP-VIEC) Measurements

The radii and content release dynamics of chromaffin vesicles were measured by RP-VIEC as previously reported.^32^ Briefly, a nanopipette (radius from 250–500 nm) filled by the vesicle suspension was placed close to a carbon fiber microdisk electrode. The vesicle suspension was prepared by diluting isolated vesicles in a homogenizing buffer with osmolarity (∼320 mOsm/kg) close to that of the intravesicular lumen to avoid the swelling and rupture of vesicles. A pressure pulse (0.5–1 s) was applied inside the nanopipette to push out the vesicle suspension onto a carbon fiber electrode and push away the bath solution, with a lower osmolarity (∼150 mOsm/kg), surrounding the electrode. Then the pressure pulse was stopped (3–5 s), and the surrounding bath solution was returned to the electrode to stop new vesicles from flowing out owing to the inward capillary force of nanopipette. The low osmolarity of the surrounding bath solution facilitated the electroporation of vesicles attached to the electrode surface. This pressure procedure was periodically applied to target vesicles one at a time onto the electrode.

Two amplifiers (Axopatch 200B, Molecular Devices, Sunnyvale, CA) were used for synchronous recording of the resistive pulses via the nanopipette and current spikes with a carbon fiber microdisk electrode as the VIEC electrode. For the RP measurement, the electrode potential was set at −13 mV through a silver wire inside the nanopipette versus a Ag/AgCl reference electrode in the external bath solution. The resistive pulse was recorded and further converted into the particle size based on the algorithm developed by Gyurcsányi and co-workers, who also calibrated the measurement.^49^ For the VIEC measurements, a beveled carbon fiber disk electrode was used to monitor the vesicular content, and its potential was set at +700 mV versus the same Ag/AgCl reference electrode used in the above RP test. Only one RP signal and one VIEC spike at one pressure cycle were collected and considered to come from the same vesicle.

### Finite Element Simulation

Although the vesicle is speculated to form a contacting area and deform in this work, severe deformation is still unexpected because of the high membrane tension as discussed in the main text. The geometry of vesicle is assumed as a sphere during the attachment and transient release. The approach to the finite element simulation of current spikes from vesicular release has been discussed in general in previous work.^24,29,30,36^ A 2-D symmetrical geometric model, defining the diffusion space of vesicular contents, was built as depicted in Figure S1.

At *t* = 0 ms, the circular region defined the vesicle interior and its radius (*R*_ves_) was calculated from the RP signal of each vesicle. The initial pore radius was set at 10^–10^ m to avoid a high initial outflow of catecholamine. The pore length was 5 nm which is close to the vesicle membrane thickness. A distance between the pore bottom to the electrode surface was set as 20 nm to describe the space formed by the membrane protein pushing the membrane away from electrode surface. The distances were set as such to avoid the overfine meshing which leads a significant calculation time. A series of simulated current curves of different distances (2–20 nm) were compared and almost overlapped (see Figure S4) so this distance does not affect our calculation within this range. The diameter of electrode surface was depicted as 5 μm,^30^ which is enough to electrooxidize all released catecholamines before they diffuse away.

The initial concentration (*C*_0_) of catecholamine within vesicle was calculated by
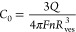
3where *Q* is the total charge of the current spike from each single vesicle VIEC measurement, *n* is the number of electron transfer (2 for catecholamines), and *F* is the Faraday constant (96485 C/mol). The initial concentration in the pore and the domain outside the vesicle was 0 mM. The boundary of the domain was impermissible except the catecholamine concentration of electrode surface was fixed at 0 M.

After *t* = 0 s, the pore radius was dynamic and defined by an interpolation function (*R*_p_(*t*), see its calculation in next section). A moving mesh module was adopted to facilitate the simulation of the pore dynamic extension. With the pore opening, the vesicle will entirely move downward and the bottom part will merge into the gap space between vesicle and electrodes. This loss of material will not lead to an error in the calculation because most values of *R*_pore_/*R*_ves_ in this work are <0.3, i.e., the angular aperture <19.5°. Within this range, the lost volume ratio to the spherical vesicle is only <0.8%. So the geometry changing during the pore opening in the simulation should not be significant.

The diffusion of catecholamine was calculated by Fick’s second law
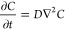
where *C* is the concentration of catecholamine, *D* is diffusion coefficient and separately set as *D*_in_ (within the vesicle, 6.0 × 10^–11^ m^2^/s),^50^ and *D*_out_ (within the pore and outside the vesicle, 6.0 × 10^–10^ m^2^/s).^51^

The surface integration of normal flux of catecholamine across the pore was calculated and converted into the simulated current (*I*_sim_), as described by
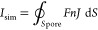
4where *J* is the flux density of catecholamine across the vesicle pore and *S*_pore_ is the pore area. See more configuration details in the Supporting Information.

### Reconstruction of Pore-Opening Dynamics

The reconstruction of the pore radius change over time (*R*_p_(*t*)) was based on searching the pore radius whose simulated current (*I*_sim_(*t*)) fits best to the experimental current data *I*_exp_(*t*) at each time point. The experimental current spike, as the objective, was intercepted from the amperometric trace, and its baseline was subtracted. After the *R*_ves_ was determined by RP measurement, the finite element model with the *R*_ves_ and corresponding initial concentration (*C*_0_) was constructed as the initial condition (*t* = 0 ms) following the above finite element model.

At *t* = *n* × Δ*T* (Δ*T* = 0.1 ms), the *R*_p_(*t*) was set as a linear interpolation function defined by a series of discrete radius values
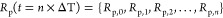
with the time series {0 ms, 0.1 ms, 0.2 ms, ..., *n* × Δ*T*} as their arguments.

Each value of *R*_p,*n*_ was estimated in sequence. The first value of *R*_p_, i.e., *R*_p,0_, was set as 10^–10^ m. Then the estimation of the *R*_p,1_ was based on searching for the *R*_p,1_ that had the least difference between the experimental current (*I*_exp_(1 × Δ*T*)) and the corresponding simulated current (*I*_sim,1_) with the Nelder–Mead method.^37,38^ After the value of *R*_p,1_ was determined, we set *R*_p,0_ ∼ *R*_p,1_ as the known parameters and continued to estimate the *R*_p,2_ by the same approach as for *R*_p,1_. Hence, after each data point (*R*_p,0_ ∼ *R*_p,t(*n*-1)_) was determined, the value of *R*_p,*n*_ was estimated, and eventually all values of *R*_p_ were obtained. For each estimation, the search zone of *R*_p,*n*_ was set to range from 10^–11^ m to 0.8 × *R*_ves_ and the optimal tolerance of argument was set as 10^–11^ m. See the schematic in Figure S5.

## Supporting Information Available

Experimental details: list of chemicals and solutions; preparation protocol of chromaffin vesicle, liposome, and carbon fiber electrode, nanopipette; configuration of RP-VIEC recording and the data processing and statistical analysis; theoretical analysis of pore size on a biovesicle membrane. Configurations of the vesicular release simulation: parameters, definitions, geometry, moving mesh configuration, coefficient from PDE, transport of diluted species, meshes, study supplemental images: schematic, essential parameters and simulated concentration profile of vesicular release on a flat electrode surface (Figure S1); four examples of experimental spikes and their best-fit *R*_p_(*t*) calculated by the FESA (Figure S2); correlation between the ratio of pore radius maxima (*R*_p,max_) to vesicle radius (*R*_ves_) and the vesicle radius (Figure S3); simulated currents of different electrode-pore distances (Figure S4); schematic of the protocol for estimating the pore radius by the FESA (Figure S5) (PDF)
Movie showing the finite element simulation result of vesicular release via a pore at the electrode surface (MP4)
