# Supplementary material for: Pore-Opening Dynamics of Single Nanometer Biovesicles at an Electrified Interface
Source: ACS Nano. 2022 Jun 1;16(6):9852–8. doi: 10.1021/acsnano.2c03929 (PMC9245343; doi:10.1021/acsnano.2c03929)
Supplement: Supplementary file 1 — nn2c03929_si_001.pdf [file nn2c03929_si_001.pdf]

Supporting Information

Pore Opening Dynamics of Single Nanometer Biovesicles at an Electrified Interface

Xinwei Zhang, Andrew G. Ewing*

Department of Chemistry and Molecular Biology, University of Gothenburg, SE-412 96 Gothenburg, Sweden

*Corresponding Author E-mails: andrew.ewing@chem.gu.se

CONTENTS

EXPERIMENTAL DETAILS

Chemicals
Solutions
Chromaffin Vesicle Preparation
Liposome preparation
Carbon Fiber Microdisk Electrode Fabrication
Nanopipette Fabrication
RP-VIEC Recording Configuration
Data Processing and Statistics
Theoretical Analysis of Pore Size

Deatails of the configurations of the vesicular release simulation

Parameters
Definitions
Geometry
Moving Mesh Configuration
Coefficient Form PDE
The Transport of Diluted Species
Meshes
Study

SUPPLEMENTAL IMAGES

Figure S1. Schematic, essential parameters and simulated concentration profile of vesicular release on a flat electrode surface.
Figure S2. 4 Examples of experimental spikes and their best-fit $R_p(t)$ calculated by the FESA.
Figure S3. The correlation between the ratio of pore radius maxima ($R_{p,max}$) to vesicle radius (R_{ves}) and the vesicle radius.
Figure S4. The simulated currents of different electrode-pore distances.
Figure S5. The schematic of the protocol for estimating the pore radius by the FESA.

REFERENCES

EXPERIMENTAL DETAILS

Chemicals

All chemicals were of analytical grade and obtained from Sigma-Aldrich, Sweden, unless otherwise stated. The chemicals were used as received without other treatments. All solutions were prepared using 18 $M\Omega cm^{-1}$ water from Purelab Classic purification system (ELGA, Sweden).

Solutions

Lock's buffer for glands storage:

1.54 M NaCl, 56 mM KCl, 36 mM $NaHCO_3$, 56 mM glucose and 50 mM HEPES with pH 7.4 was diluted 10 times with distilled water.

Homogenizing buffer for vesicle storage:

0.3 M sucrose, 1 mM EDTA, 1 mM $MgSO_4$, 10 mM HEPES, 10 mM KCl and cOmplete Protease Inhibitor obtained from Roche was used for tissue homogenization and following amperometric recording. The solution should be adjusted to pH 7.4 and its osmolality ~320 mOsm/kg.

Bath solution for VIEC measurements:

3 mM D-glucose, 20 mM HEPES, 1.2 mM KH_2PO_4, 1.2 mM $MgSO_4$, 2.4 mM $CaCl_2$, 58 mM NaCl, 60 mM KCl. The osmolality was ~150 mOsm/kg and 1% v/v DMSO was added to improve the vesicle breaking efficiency.

Lipid film hydration buffer for preparing liposomes:

150 mM dopamine (DA) hydrochloride in 10 mM HEPES at pH 7.4. It was purged with argon for 30 min before adding oxygen sensitive DA hydrochloride.

Pre-equilibration buffers for the Sephadex G-25 column and Liposome isotonic buffer for the storage, amperometric measurements:

142 mM NaCl in 10 mM HEPES with pH 7.4.

Chromaffin Vesicle Preparation

Chromaffin vesicles were isolated according to our previously reported procedure[1,2] which was developed by the Borges laboratory (Unversidad de La Laguna, Tenerife, Spain). Bovine adrenal glands were obtained from a local slaughterhouse.

Briefly, after cutting off the outer fat and cleaning internal blood of adrenal glands with Lock’s buffer, we separated the medulla with a surgical scissor and mechanical homogenized in ice-cold homogenizing buffer. Then the dispersion was centrifuged at

1000×g for 10 min at 4°C, the supernatant collected, and centrifuged again at 10000×g for 20 min at 4°C to obtain pellet chromaffin vesicles. The pellet was re-suspended in 1 mL homogenizing buffer and used as vesicle stock solution.

Liposome preparation

Neurotransmitter-loaded liposomes were prepared passively by thin lipid film hydration. A solution of 1,2-dioleoyl-*sn-glycero*-3-phosphocholine (DOPC), 1,2-dioleoyl-*sn-glycero*-3-phosphethanolamine (DOPE) and cholesterol (60:20:20 mole ratio) in chloroform was dried in a round-bottom flask by rotary evaporation until a lipid film was obtained (~3 h). The dried lipid cake was re-hydrated to obtain a liposome suspension by gently mixing with 1.5 mL of the hydration buffer and then left to stand for 30 min under argon gas at room temperature. The liposome suspension was freeze-thawed in liquid nitrogen 3 to 5 times to form multilamellar vesicles, and then extruded 11 times through double polycarbonate membranes of 0.4 μm pore size with an Avanti Mini-Extruder (Avanti Polar Lipids, Inc., USA).

Carbon Fiber Microdisk Electrode Fabrication

The microdisk electrode for VIEC was fabricated as previously described.[1] Briefly, a 33-μm diameter carbon fiber was aspirated into a borosilicate capillary (1.2 mm O.D., 0.69 mm I.D., Sutter Instrument Co., Novato, CA, U.S.A.). The capillaries were subsequently pulled with a vertical puller (Model PE-21, NARISHIGE, Japan). The fiber was fixed and the electrode was sealed by dipping the pulled tip in epoxy (Epoxy Technology, Billerica, MA, U.S.A). The glued electrodes were cured in an oven at 100°C overnight and subsequently cut at the glass junction and beveled at 45° angle (EG-400, Narishige Inc., London, UK). Prior to use for VIEC, the electrode responses were tested by performing cyclic voltammetry (-0.2 to 0.8 V vs. Ag/AgCl, at 100 mV/s) in a solution of 100 μM dopamine in PBS (pH 7.4).

Nanopipette Fabrication

The nanopipette for RP recording was fabricated with a laser puller (Model P-2000, Sutter Instruments Co., Novato, CA, U.S.A.) to pull borosilicate glass capillaries (1.0 mm O.D., 0.78 mm I.D., Sutter Instrument Co., Novato, CA, U.S.A.). The radii varied from 250~500 nm measured by scanning electron microscopy.

RP-VIEC Recording Configuration

The RP-VIEC recording was synchronously performed by two sets of amplifiers (Axopatch 200B, Molecular Devices, Sunnyvale, CA, U.S.A.). They shared the same reference electrode (Ag/AgCl).

RP recording:

0.1 mL vesicle suspension was diluted into 1.0 mL homogenizing buffer, filtered by 0.45 μm filter (Cellulose acetate membrane, VWR, Sweden) and injected into the nanopipette. Then the nanopipette was connected to a microinjector system (Femtojet, Eppendorf Co., Germany) with a holder, in which a platinum wire was placed. Then the holder was connected to an amplifier (the work potential was set at +13 mV). The microinjector conducted the pressure which was set at 0.5~1 s, 50~100 hPa, without compensation pressure. The pressure value and time were adjusted based on the RP amount during each pushing stage. The nanopipette was placed at ~45° to the carbon fiber microdisk electrode surface plane in bath solution, and the distance was ~10 μm.

VIEC recording:

The carbon fiber microdisk electrode was placed in the bath solution and connected to another amplifier (the working potential was set at +900 mV). The recorded RP and VIEC signals were filtered at 2 kHz using a 4-pole Bessel filter and digitized at 10 kHz using a Digidata model 1440A with Axoscope 10.3 software.

Data Processing and Statistics

All RP and VIEC data were analyzed by a series of home-built programs in MATLAB (MathWorks Inc., Natick, MA, U.S.A.). Due to the periodic pulse, the baseline of the RP and VIEC traces both increased and decreased in cycles.

RP signals:

The conversion from RP signal to size of vesicle was carried out based on the algorithm reported by Gyurcsányi and coworkers.[3] Briefly, the RP raw traces were normalized based on the nearby baseline and converted into percentage ratio ($\Delta I/I$). Then RP signals were searched and counted only in the pushing stage in each cycle, and the detection limit was 5 times the standard deviation of the baseline noise in the pushing stage. Then, the normalized RP signals ($\Delta I/I$) were converted into normalized resistance changes (ΔR^*, see equations 4/5 in Ref [3]) and the radius ratio of vesicle to nanopore. After we measured the size of nanopore by SEM, the radius of vesicle was obtained.

VIEC signals:

After subtracting the baseline of a VIEC raw trace, the VIEC current spikes were searched and counted in each cycle. The detection limit used was 6 times the standard deviation of the baseline noise in a whole cycle. The area of each spike was the total charge transferred from the released catecholamine to the electrode. The vesicular content in moles was obtained by use of Faraday’s Law, in which the charge transfer number of electrons is 2. All traces were visually inspected, and false positives were removed.

Theoretical Analysis of Pore Size

As discussed in the main text, the maximal pore area, $S_{\mathrm{p,max}}$ is the addition of the areas of the small membrane sections those that were electroporated, *i.e.,*

$S_{\mathrm{p,max}} = \sum_{i=1}^{n_{\mathrm{e}}} A_{\mathrm{s},i}$

where the A_{s} is the area of each small section and $n_{\mathrm{e}}$is the amount of the electroporated sections. If considering the probability of electroporation occuring in each respective small section (P_{a}). The formula can be written as,

$$S_{\mathrm{p,max}} = \sum_{i=1}^{n_{\mathrm{a}}} A_{\mathrm{s},i} P_{\mathrm{a},i}$$

where n_{a} is the total amount of the small sections within the contact area. Within each small membrane section, we can simplify the section as a pure lipid membrane. Thus, based on the previous discusses on the electroporation on a lipid sheet,[4] the electroporation probability of each small section is related to the area of the section, expressed as,

$$P_{\mathrm{a}} = A_{\mathrm{s}} p_{\mathrm{e}}$$

where the p_{e} is the irreversible electroporation probability of a small element of vesicular membrane and can be considered as a constant within the contact area of any vesicles, because of the similar electric field across the similar vesicular membrane thickness even at different sized vesicles. So, the $S_{\mathrm{p,max}} = p_{\mathrm{e}} \sum_{i=1}^{n_{\mathrm{a}}} A_{\mathrm{s},i}^2$, which can be expressed in the form of average, $S_{\mathrm{p,max}} = p_{\mathrm{e}} n_{\mathrm{a}} \overline{A_{\mathrm{s}}^2}$. Considering the uniform distribution of membrane proteins, $\overline{A_{\mathrm{s}}^2} \approx \overline{A_{\mathrm{s}}}^2$, we could conclude that the $S_{\mathrm{p,max}} = p_{\mathrm{e}} n_{\mathrm{s}} \overline{A_{\mathrm{s}}}^2$.

SUPPLEMENTAL IMAGES

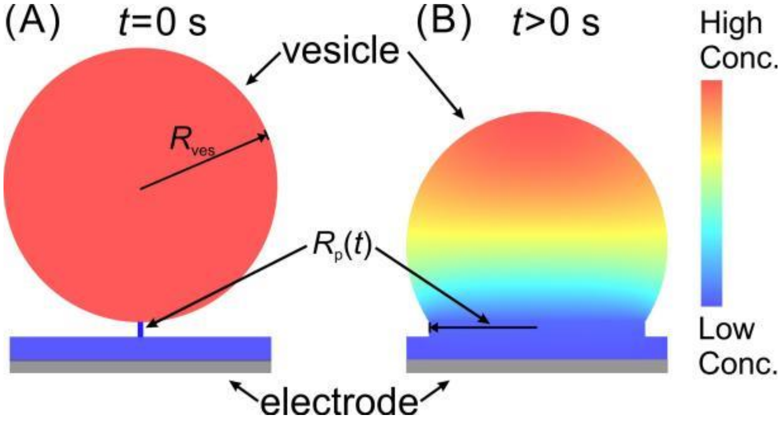

Figure S1. Schematic, essential parameters and simulated concentration profile of vesicular release on a flat electrode surface. This model describes the initial high concentration of catecholamine within vesicle lumen (A, *t*=0 ms) and its gradient formed after the pore expands (B, *t*>0 ms). The R_{ves} is the vesicle radius obtained by resistive pulse (RP) measurement; the $R_p(t)$ presents the pore radius change over time. The concentration of catecholamine at electrode surface was set at 0 mM. Picture is not drawn according to scale.

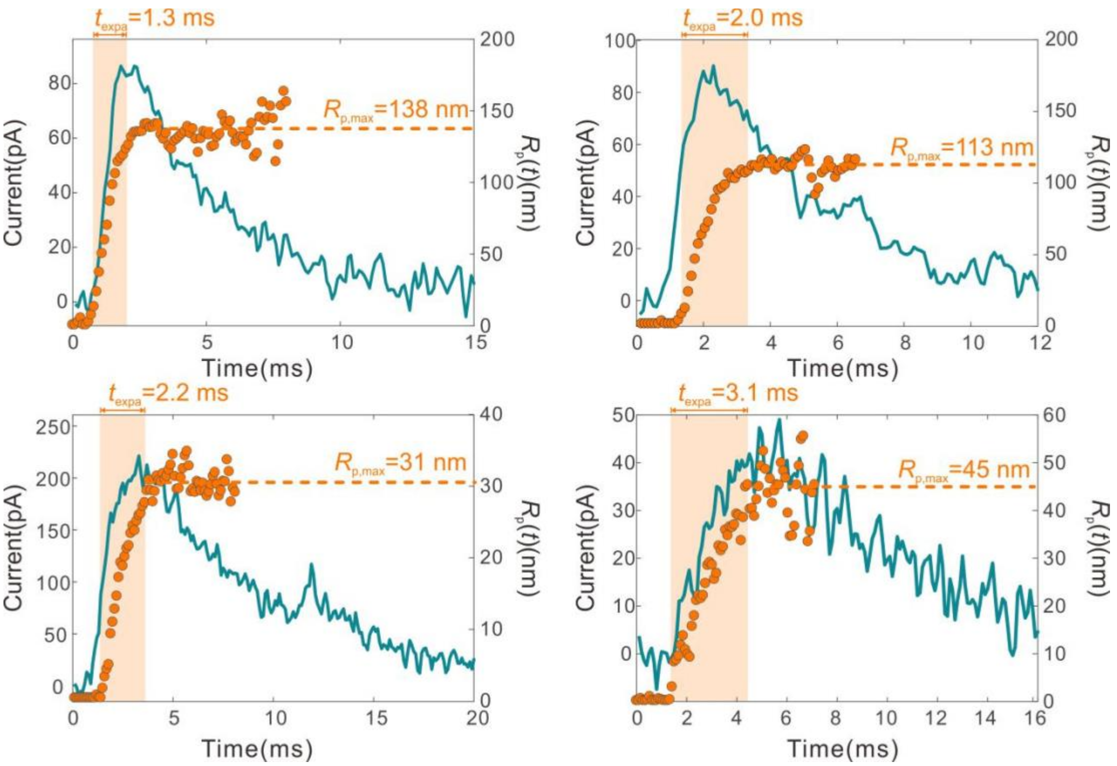

Figure S2. 4 Examples of experimental spikes and their best-fit $R_p(t)$ calculated by the FESA. The experimental spikes are drawn as blue line and associated to the left-Y axis. The best-fit $R_p(t)$ are drawn as orange dots and associated to the right-Y axis. The time of pore expansion from 5% to 95% of its maximum (t_{expa}) is indicated as a yellow zone.

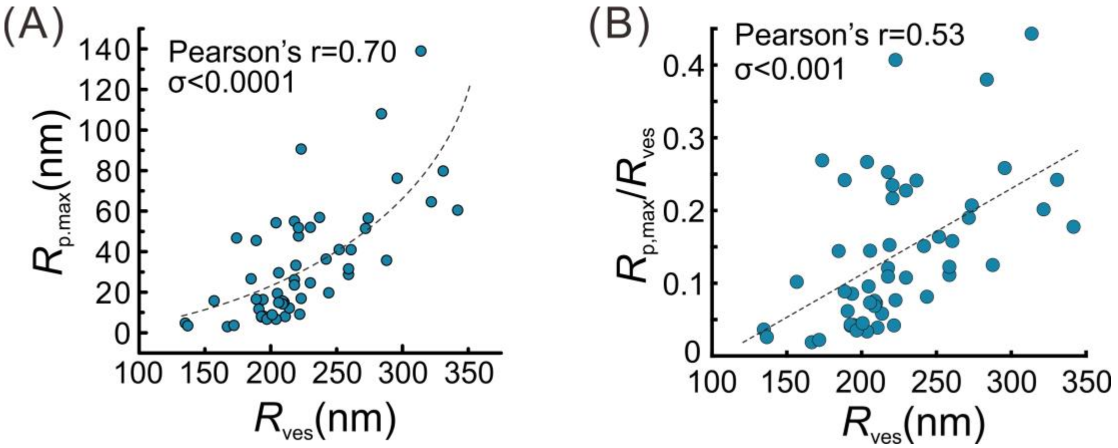

Figure S3. The correlation between (A) the $R_{p,max}$ and (B) the ratio of pore radius maxima ($R_{p,max}$) to vesicle radius (R_{ves}) and the vesicle radius. A Pearson's test was applied to evaluate the correlation. The expression of fitting curve in left figure is $R_{p,max}=2.3\times10^{-7}R_{ves}^{3.43}$ (unit: nm).

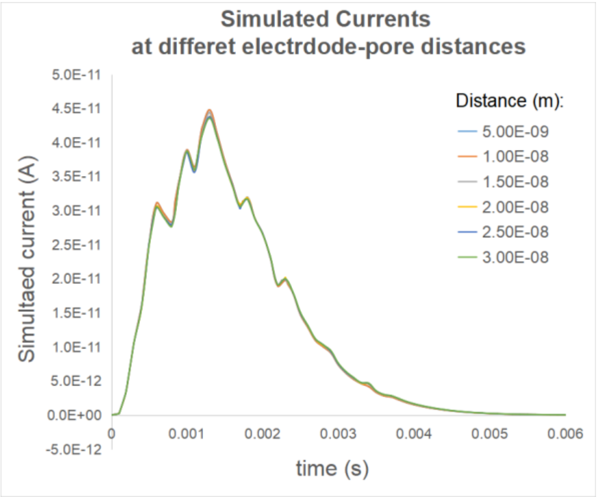

Figure S4. Simulated currents of different electrode-pore distances. These are mostly overlapping so that the distance effects can be ignored in this configuration.

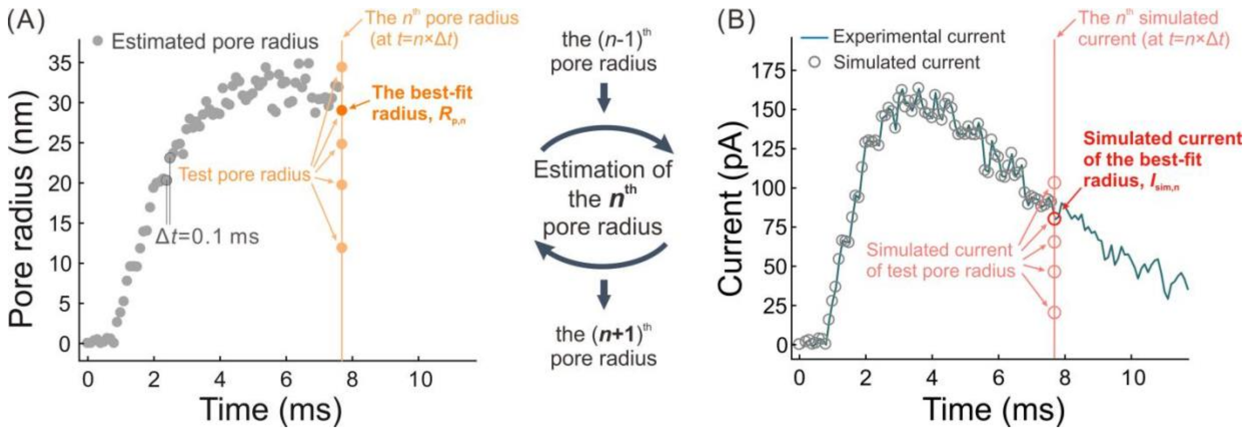

Figure S5. Protocol for estimating the pore radius at $t=n\times\Delta T$ ($\Delta T=0.1$ ms). (A) The plot of pore radius is being estimated (at $t=n\times0.1$ ms, light yellow dots and the best-fit value as a dark yellow dot) and those have been estimated (from $t=0.1$ ms to $(n-1)\times0.1$ ms, gray dots). (B) Plot of simulated currents corresponding to the estimated pore radius (gray circles), and those to the pore radius being tested (light red circles, the best-fit simulated current as a dark red circle). The best-fit value of pore radius was searched by the Nelder-Mead algorithm, whose corresponding simulated current (dark red circle) fits best to the experimental current (blue line). This protocol was repeated from $t=0.1$ ms to the end of the experimental spike.

Details of the configurations of the vesicular release simulation

Calculations were carried out on a commercial finite elements simulation software - COMSOL Multiphysics 5.3. The configuration was in the format of the COMSOL interface.

The *Transport of Diluted Species* module was used to simulate catecholamine diffusion and consumption, whereas the *Moving Meshes* and the *Coefficient Form PDE* modules were used to control the deformation of vesicle geometry and re-meshing during the pore opening. Please see more discussion and mechanism about the model of vesicular release in the main text. The *Material Section* was not globally configured but the diffusion properties of catecholamines were set in the *Transport of Diluted Species* module.

1.1 PARAMETERS

- See the resource of each parameters in the Method Section in main text.

**Name**	**Value**	**Description**
Rp0	1E–10 m	initial pore radius
Rves	1E–7 m	vesicle radius
Dis	2E–8 m	distance between electrode surface and vesicle
PoreLength	5E–9 m	length of pore formed on the membrane
ElectWidth	2.5E–7 m	radius of electrode
c0	17.427 mol/m^3	initial concentration of catecholamines from example experimental data
Dout	6E–10 m^2/s	diffusion coefficient of catecholamine within vesicle
Din	6E–11 m^2/s	diffusion coefficient of catecholamine without vesicle
t_calc	20e-3 s	calculated time of vesicular release

1.2 DEFINITIONS

1.2.1 Variables

SELECTION

Geometric entity level	Entire model

Name	Expression	Unit	Description
Vp	vp(t)	m	See vp(t) in the section 1.2.2
gamma	acos((Rves + Vp)/Rves)	rad	A parameter facilitating the calculation of points location, see the section 1.5.5-14
beta	pi/2 - gamma	rad	A parameter facilitating the calculation of points location, see the section 1.5.5-14
Rpf	rpf_dis(t)	m	The pore size
Flux	Flux2Current(lineintop(tds.ntflux_c))	A	The current flux across the electrode surface, calculated by Faraday's law, see Eq.4 in the main text.
lineintop(tds.ntflux_c)	lineintop(tds.ntflux_c)	mol/s	The surface integration of normal total flux of catecholamines crossing the electrode surface (the bottom edge of r1 in the Section 1.3).

1.2.2 Functions

Function: vp(t)

Function name	vp(t)
Function type	Piecewise

DEFINITION

Description	Value
Argument	t
Extrapolation	None
Smoothing	No smoothing

DEFINITION

Start	End	Function
0	t_calc	Rves*(cos(asin(rpf_dis(t)/Rves))-1)

UNITS

Description	Value
Arguments	s
Function	m

Function: Flux2Current(c)

Function name	Flux2Current
Function type	Analytic

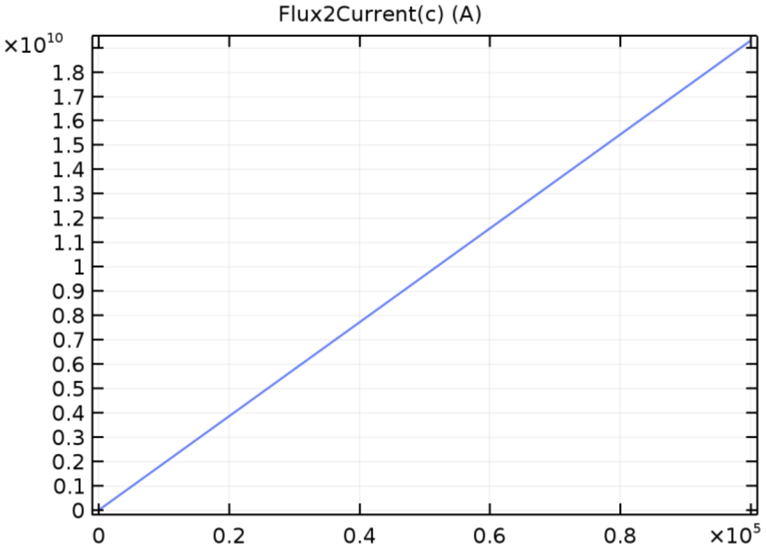

DEFINITION

Description	Value
Expression	2*F_const*c
Arguments	c

UNITS

Description	Value
Function	A

Function: rpf_dis(t)

Function name	rpf_dis
Function type	Piecewise

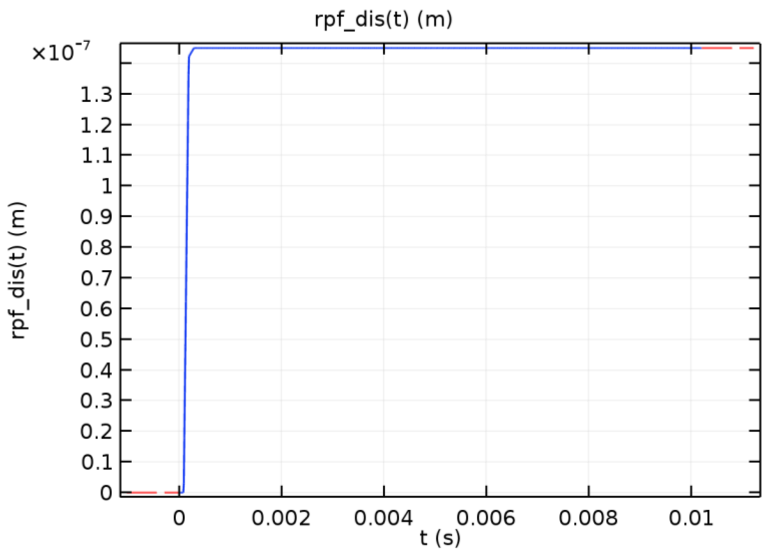

DEFINITION

Description	Value
Argument	t
Extrapolation	Constant
Smoothing	Continuous function

SETTINGS

Description	Value
Size of transition zone	0.01

DEFINITION

Start	End	Function
0	t_calc	rpf_dis_table(t)

UNITS

Description	Value
Arguments	s
Function	m

Function: rpf_dis_table(t)

- The pore radius table, i.e. $R_p(t)$ obtained by the reconstruction algorithm in Method Sections in the main text.

Function names	rpf_dis_table
Function type	Interpolation

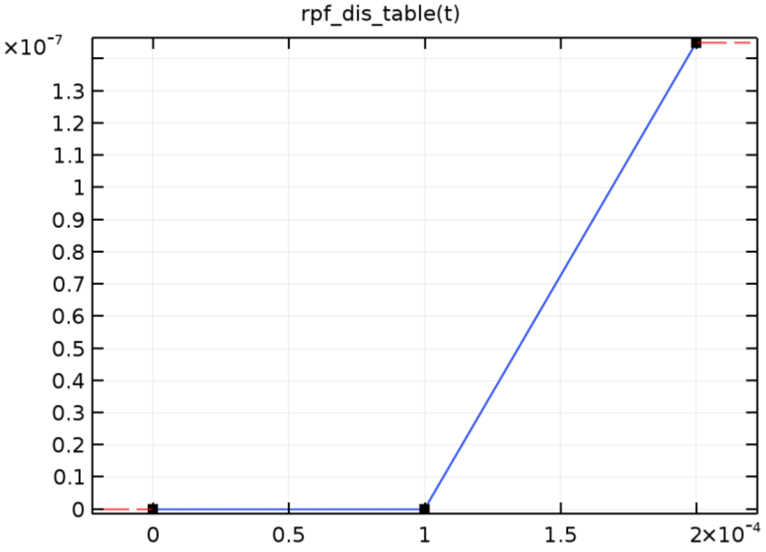

1.2.3 Coordinate Systems

COORDINATE NAMES

First	Second	Third
t1	to	n

SETTINGS

Description	Value
Frame	Reference configuration

1.3 GEOMETRY

- This model was constructed in a 2-D axisymmetric space, scalar field.
- The symmetric axis is labeled by a red dashed line.
- The rectangle (r3) is a domain generated to avoiding the separation of Pore zone (r2) and Vesicle domain (c1) during pore opening. Its geometry is the intersection of r2 and c1.

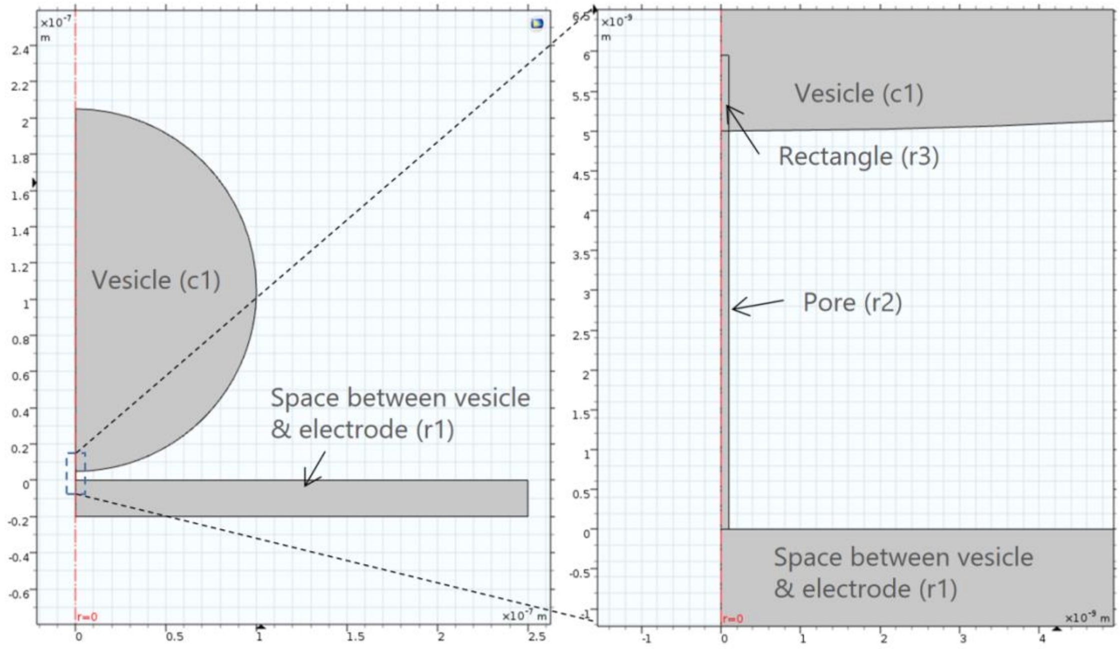

UNITS

Length unit	m
Angular unit	deg

1.3.1-3 Domains:

1.3.1 Space between vesicle & electrode (r1)

POSITION

Description	Value
Position	{-ElectWidth/2, -Dis}

SIZE

Description	Value
Width	ElectWidth
Height	Dis

1.3.2 Vesicle (c1)

POSITION

Description	Value
Position	{0, Rves + PoreLength}

SIZE AND SHAPE

Description	Value
Radius	Rves

1.3.3 Pore (r2)

POSITION

Description	Value
Position	{0, 0}

SIZE

Description	Value
Width	Rp0
Height	PoreLength + 0.01*Rves

1.3.4-14 The points on the edge of vesicle domain (c1)

- These points were set to facilitate the mesh moving smoothly
- a=π/24 as defined in the Section 1.2

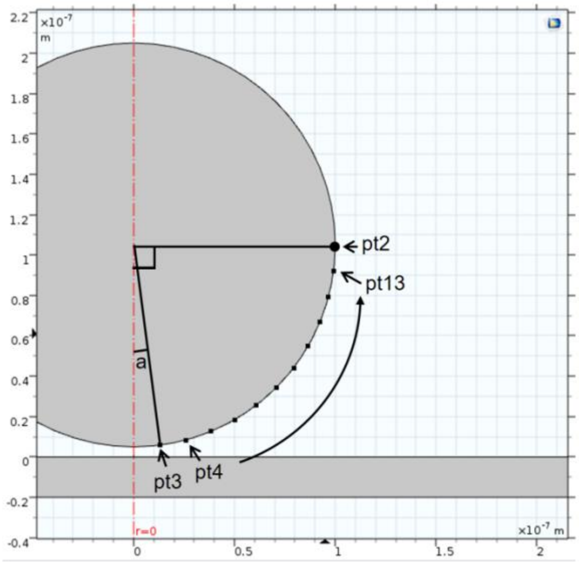

1.3.4 point 3 (pt3)

POINT

Description	Value
Point coordinate	{sin(a)*Rves, Rves + PoreLength - cos(a)*Rves}

1.3.5 point 4 (pt4)

POINT

Description	Value
Point coordinate	{sin(2*a)*Rves, Rves + PoreLength - cos(2*a)*Rves}

1.3.6 point 5 (pt5)

POINT

Description	Value
Point coordinate	{sin(3*a)*Rves, Rves + PoreLength - cos(3*a)*Rves}

1.3.7 point 6 (pt6)

POINT

Description	Value
Point coordinate	{sin(4*a)*Rves, Rves + PoreLength - cos(4*a)*Rves}

1.3.8 point 7 (pt7)

POINT

Description	Value
Point coordinate	{sin(5*a)*Rves, Rves + PoreLength - cos(5*a)*Rves}

1.3.9 point 8 (pt8)

POINT

Description	Value
Point coordinate	{sin(6*a)*Rves, Rves + PoreLength - cos(6*a)*Rves}

1.3.10 point 9 (pt9)

POINT

Description	Value
Point coordinate	{sin(7*a)*Rves, Rves + PoreLength - cos(7*a)*Rves}

1.3.11 point 10 (pt10)

POINT

Description	Value
Point coordinate	{sin(8*a)*Rves, Rves + PoreLength - cos(8*a)*Rves}

1.3.12 point 11 (pt11)

POINT

Description	Value
Point coordinate	{sin(9*a)*Rves, Rves + PoreLength - cos(9*a)*Rves}

1.3.13 point 12 (pt12)

POINT

Description	Value
Point coordinate	{sin(10*a)*Rves, Rves + PoreLength - cos(10*a)*Rves}

1.3.14 point 13 (pt13)

POINT

Description	Value
Point coordinate	{sin(11*a)*Rves, Rves + PoreLength - cos(11*a)*Rves}

1.4 MOVING MESH CONFIGURATION

- The moving mesh is realized by the combination of *Moving Meshes* module and the *Coefficient Form PDE* Interface (see section 1.5). Their specific configurations are listed below according to the format of COMSOL Multiphysics 5.3.
- *Vp* is a variable defined in section 1.2, which is used to control and coordinate the moving speed of vesicle and the pore.
- The controlled domain or boundary of each configuration is marked as blue.

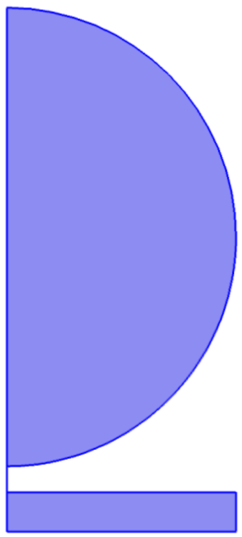

Moving Mesh

SELECTION

Geometric entity level	Domain
Selection	All Domains

SETTINGS

**Description**	**Value**
Geometry shape order	1
Use legacy moving frame functionality	Off

1.4.1 Free Deformation 1

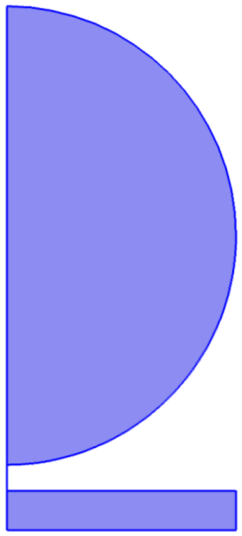

Free Deformation 1

SELECTION

Geometric entity level	Domain
Selection	All Domains

SETTINGS

Description	Value
Initial mesh displacement	{0, 0}

1.4.2 Prescribed Mesh Displacement 2

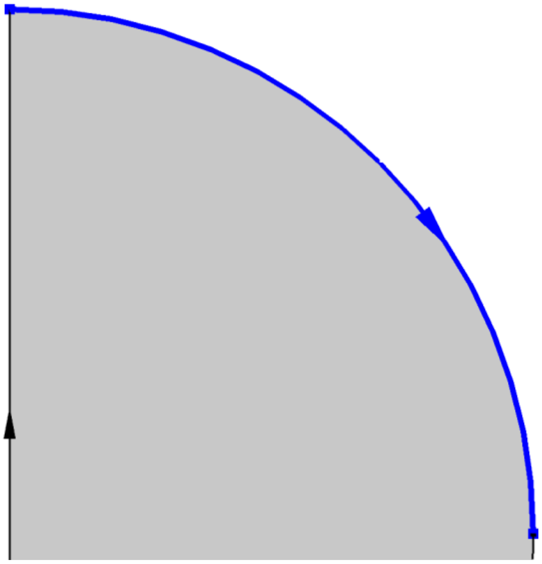

Prescribed Mesh Displacement 2

SELECTION

Geometric entity level	Boundary
Selection	Edge of upper semicircle of c1

SETTINGS

**Description**	**Value**
Prescribed # displacement	{On, On}
Prescribed mesh displacement	{0, Vp}
Use weak constraints	Off

1.4.3 Prescribed Mesh Displacement 3

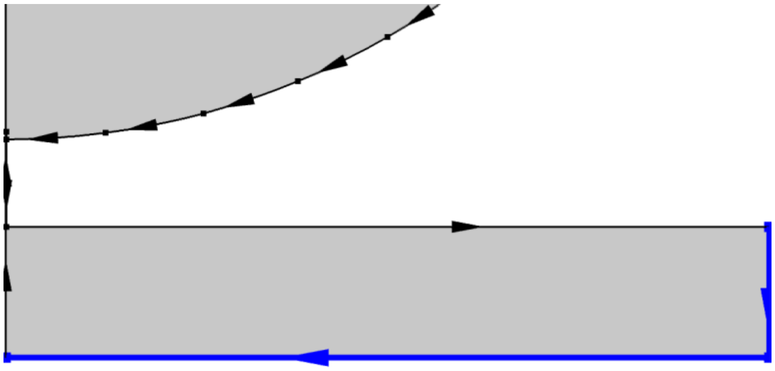

Prescribed Mesh Displacement 3

SELECTION

Geometric entity level	Boundary
Selection	Bottom and right boundary of r1

SETTINGS

**Description**	**Value**
Prescribed # displacement	{On, On}
Prescribed mesh displacement	{0, 0}
Use weak constraints	Off

1.4.4 Prescribed Mesh Displacement 4

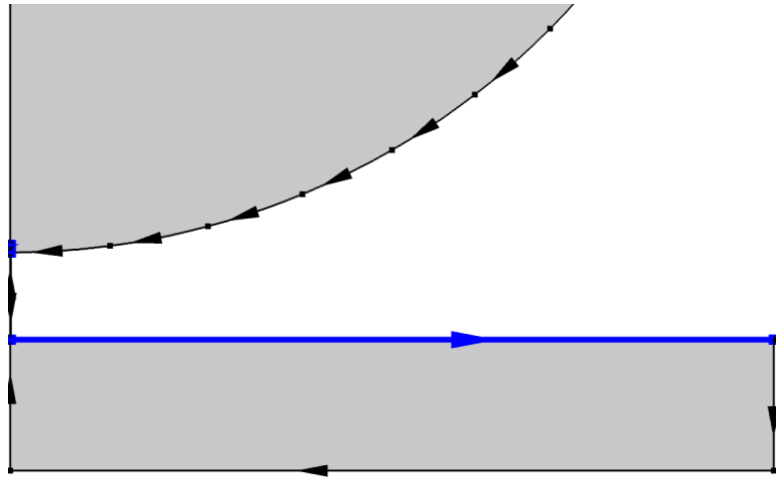

Prescribed Mesh Displacement 4

SELECTION

Geometric entity level	Boundary
Selection	Upper boundary of r1,<br>Right boundary of r2 and r3

SETTINGS

Description	Value
Prescribed # displacement	{Off, On}
Use weak constraints	Off

1.4.5 Prescribed Mesh Displacement 5

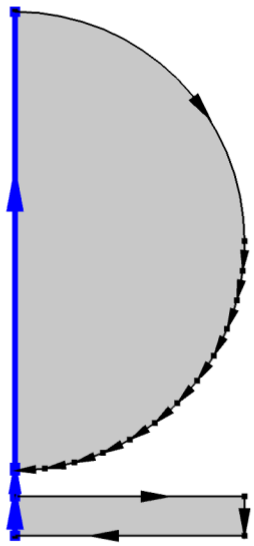

Prescribed Mesh Displacement 5

SELECTION

Geometric entity level	Boundary
Selection	The symmetric axis

SETTINGS

**Description**	**Value**
Prescribed # displacement	{On, Off}
Prescribed mesh displacement	{0, 0}
Use weak constraints	Off

1.4.6 Prescribed Mesh Displacement 7

- The values {Rhelp, Zhelp} were set as dependent variables in the *Coefficient Form PDE* interface (see the Section 1.5), which were used to describe the movement of the points on the edge of lower semicircle of c1 (the deformation of vesicle during pore opening) and the right edge of r2, r3 (the wall of pore).

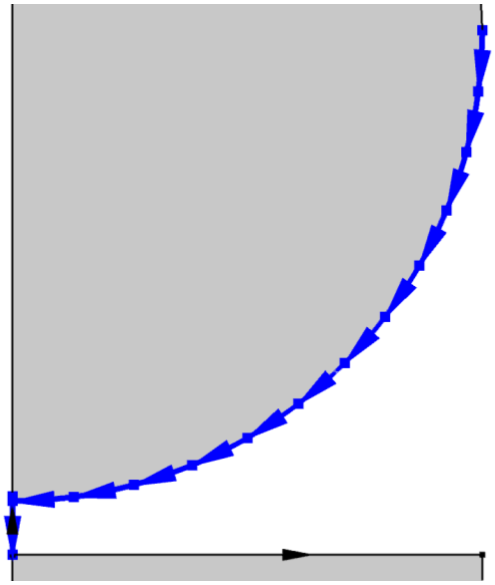

Prescribed Mesh Displacement 7

SELECTION

Geometric entity level	Boundary
Selection	Edge of lower semicircle of c1<br>Right edge of r2, r3

SETTINGS

**Description**	**Value**
Prescribed # displacement	{On, On}
Prescribed mesh displacement	{Rhelp, Zhelp}
Use weak constraints	Off

1.5 COEFFICIENT FORM PDE

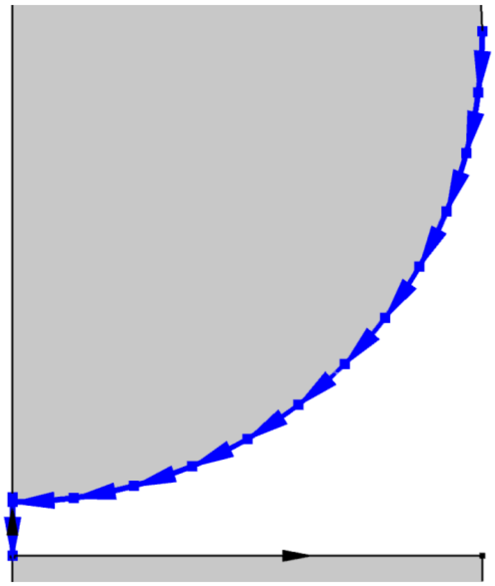

Coefficient Form PDE

SELECTION

Geometric entity level	Boundary
Selection	Edge of lower semicircle of c1<br>Right edge of r2, r3

SETTINGS

**Description**	**Value**
Shape function type	Lagrange
Element order	Quadratic
Value type when using splitting of complex variables	Complex
Frame	Spatial
Dependent variable quantity	Displacement field (m)
Source term quantity	Dimensionless (1)

1.5.1 Coefficient Form PDE 1

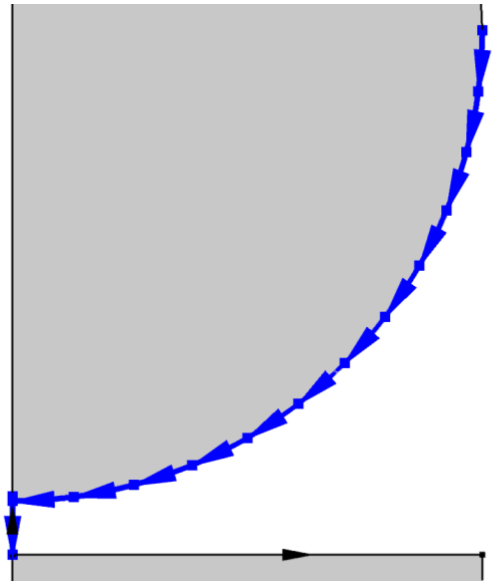

Coefficient Form PDE 1

SELECTION

Geometric entity level	Boundary
Selection	Edge of lower semicircle of c1<br>Right edge of r2, r3

EQUATIONS

$$e_a\frac{\partial^2\mathbf{u}}{\partial t^2}+d_a\frac{\partial\mathbf{u}}{\partial t}+\nabla\cdot(-c\nabla\mathbf{u}-\alpha\mathbf{u}+\gamma)+\beta\cdot\nabla\mathbf{u}+a\mathbf{u}=f$$

$$\mathbf{u}=[Rhelp, Zhelp]^T$$

$$\nabla=[\frac{\partial}{\partial r},\frac{\partial}{\partial z}]$$

SETTINGS

Description	Value
Diffusion coefficient	{{{{1, 0}, {0, 1}}, {{0, 0}, {0, 0}}}, {{{0, 0}, {0, 0}}, {{1, 0}, {0, 1}}}}
Absorption coefficient	{{0, 0}, {0, 0}}
Source term	{0, 0}
Mass coefficient	{{0, 0}, {0, 0}}
Damping or mass coefficient	{{0, 0}, {0, 0}}
Conservative flux convection coefficient	{{{0, 0}, {0, 0}}, {{0, 0}, {0, 0}}}
Convection coefficient	{{{0, 0}, {0, 0}}, {{0, 0}, {0, 0}}}
Conservative flux source	{{0, 0}, {0, 0}}

Shape functions

Name	Shape function	Unit	Description	Shape frame	Selection
Rhelp	Lagrange (Quadratic)	m	Dependent variable Rhelp	Spatial	Edge of lower semicircle of c1<br>Right edge of r2, r3
Zhelp	Lagrange (Quadratic)	m	Dependent variable Zhelp	Spatial	Edge of lower semicircle of c1<br>Right edge of r2, r3

1.5.2 Initial value 1

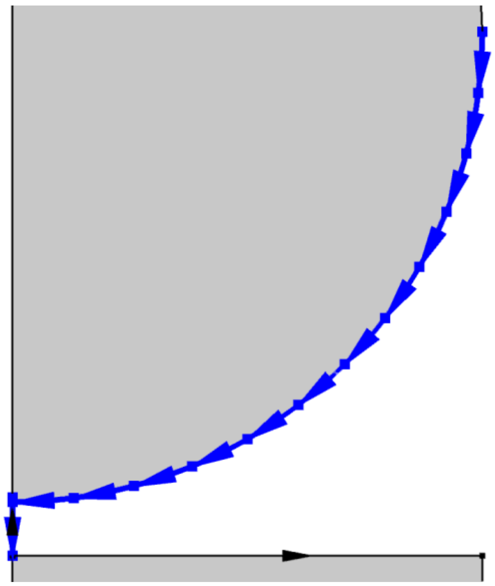

Initial value 1

SELECTION

Geometric entity level	Boundary
Selection	Edge of lower semicircle of c1<br>Right edge of r2, r3

SETTINGS

Description	Value
Initial value for Rhelp	0
Initial time derivative of Rhelp	0
Initial value for Zhelp	0
Initial time derivative of Zhelp	0

1.5.3 Dirichlet Boundary Condition 1

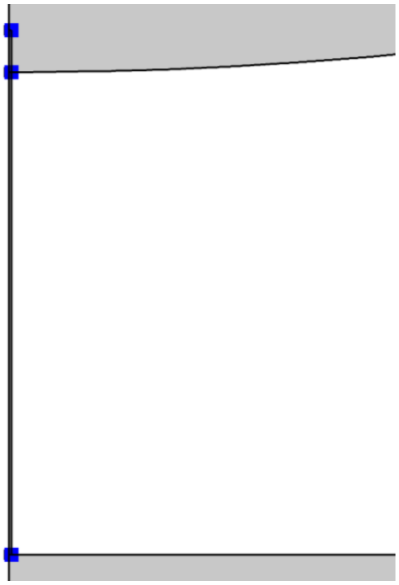

Dirichlet Boundary Condition 1

SELECTION

Geometric entity level	Point
Selection	3 Points on the right edge of r2, r3

EQUATIONS

$$\mathbf{u} = r$$
$$\mathbf{u} = [Rhelp, Zhelp]^T$$
$$g_{\mathrm{reaction}} = -\mu$$
$$\mu = [\mu_1, \mu_2]^T$$

SETTINGS

Description	Value
Value on boundary	{Rpf, 0}
Prescribed value of Rhelp	On
Prescribed value of Zhelp	On
Apply reaction terms on	Individual dependent variables
Use weak constraints	Off
Constraint method	Elemental

Shape functions

Constraint	Constraint force	Shape function	Selection
Rpf-Rhelp	-test(Rhelp)	Lagrange (Quadratic)	3 Points on the right edge of r2, r3
-Zhelp	-test(Zhelp)	Lagrange (Quadratic)	3 Points on the right edge of r2, r3

1.5.4 Dirichlet Boundary Condition 2

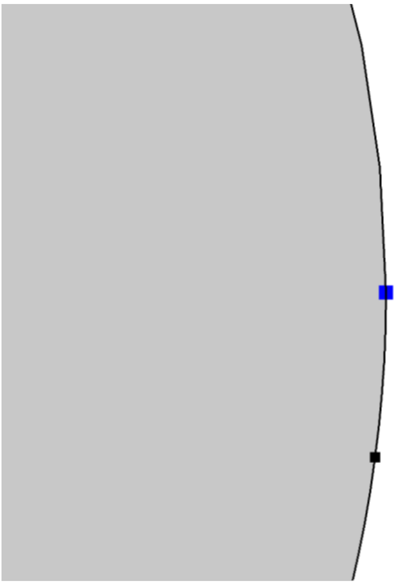

Dirichlet Boundary Condition 2

SELECTION

Geometric entity level	Point
Selection	pt2

EQUATIONS

$$\mathbf{u} = r$$
$$\mathbf{u} = [Rhelp, Zhelp]^T$$
$$g_{reaction} = -\mu$$
$$\mu = [\mu_1, \mu_2]^T$$

SETTINGS

Description	Value
Value on boundary	{0, Vp}
Prescribed value of Rhelp	On
Prescribed value of Zhelp	On
Apply reaction terms on	Individual dependent variables
Use weak constraints	Off
Constraint method	Elemental

Shape functions

Constraint	Constraint force	Shape function	Selection
-Rhelp	-test(Rhelp)	Lagrange (Quadratic)	pt2
Vp-Zhelp	-test(Zhelp)	Lagrange (Quadratic)	pt2

1.5.5 Dirichlet Boundary Condition 3

- The variable *gamma*, *beta* was defined in section 1.2

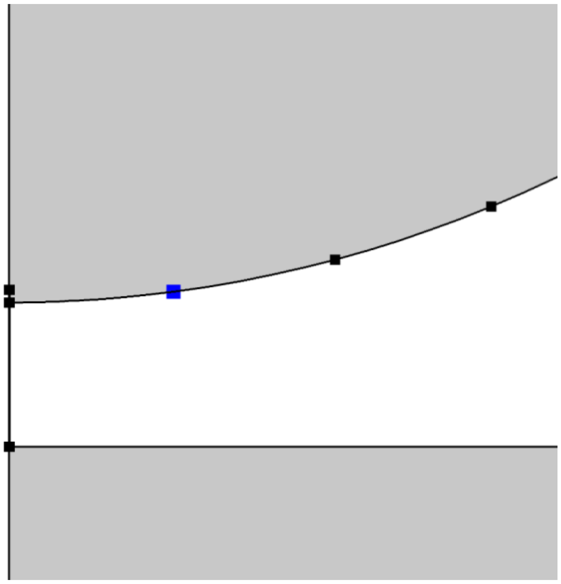

Dirichlet Boundary Condition 3

SELECTION

Geometric entity level	Point
Selection	pt3

EQUATIONS

$$\mathbf{u} = r$$
$$\mathbf{u} = [Rhelp, Zhelp]^T$$
$$g_{reaction} = -\mu$$
$$\mu = [\mu_1, \mu_2]^T$$

SETTINGS

Description	Value
Value on boundary	{Rves*sin(gamma + 1*beta/12) - Rves*sin(1*a), Vp - Rves*cos(gamma + 1*beta/12) + Rves*cos(1*a)}
Prescribed value of Rhelp	On
Prescribed value of Zhelp	On
Apply reaction terms on	Individual dependent variables
Use weak constraints	Off
Constraint method	Elemental

Shape functions

Constraint	Constraint force	Shape function	Selection
Rves*sin(gamma+beta/12)-Rves*sin(a)-Rhelp	-test(Rhelp)	Lagrange (Quadratic)	pt3
Vp-Rves*cos(gamma+beta/12)+Rves*cos(a)-Zhelp	-test(Zhelp)	Lagrange (Quadratic)	pt3

1.5.6 Dirichlet Boundary Condition 4

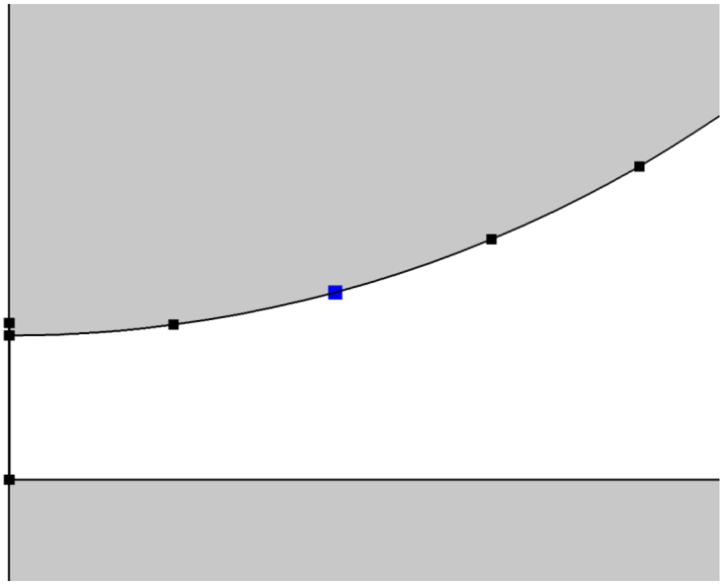

Dirichlet Boundary Condition 4

SELECTION

Geometric entity level	Point
Selection	pt4

EQUATIONS

$$\mathbf{u} = r$$
$$\mathbf{u} = [Rhelp, Zhelp]^T$$
$$g_{reaction} = -\mu$$
$$\mu = [\mu_1, \mu_2]^T$$

SETTINGS

Description	Value
Value on boundary	{Rves*sin(gamma + 2*beta/12) - Rves*sin(2*a), Vp - Rves*cos(gamma + 2*beta/12) + Rves*cos(2*a)}
Prescribed value of Rhelp	On
Prescribed value of Zhelp	On

Description	Value
Apply reaction terms on	Individual dependent variables
Use weak constraints	Off
Constraint method	Elemental

Shape functions

Constraint	Constraint force	Shape function	Selection
Rves*sin(gamma+beta/6)-Rves*sin(2*a)-Rhelp	-test(Rhelp)	Lagrange (Quadratic)	pt4
Vp-Rves*cos(gamma+beta/6)+Rves*cos(2*a)-Zhelp	-test(Zhelp)	Lagrange (Quadratic)	pt4

1.5.7 Dirichlet Boundary Condition 5

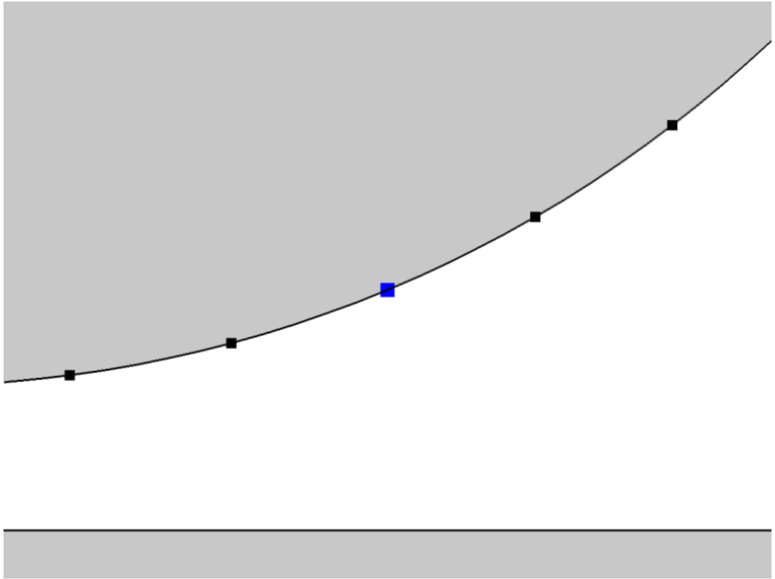

Dirichlet Boundary Condition 5

SELECTION

Geometric entity level	Point
Selection	pt5

EQUATIONS

$$\mathbf{u} = r$$
$$\mathbf{u} = [Rhelp, Zhelp]^T$$
$$g_{reaction} = -\mu$$
$$\mu = [\mu_1, \mu_2]^T$$

SETTINGS

Description	Value
Value on boundary	{Rves*sin(gamma + 3*beta/12) - Rves*sin(3*a), Vp - Rves*cos(gamma + 3*beta/12) + Rves*cos(3*a)}
Prescribed value of Rhelp	On
Prescribed value of Zhelp	On
Apply reaction terms on	Individual dependent variables
Use weak constraints	Off
Constraint method	Elemental

Shape functions

Constraint	Constraint force	Shape function	Selection
Rves*sin(gamma+0.25*beta)-Rves*sin(3*a)-Rhelp	-test(Rhelp)	Lagrange (Quadratic)	pt5
Vp-Rves*cos(gamma+0.25*beta)+Rves*cos(3*a)-Zhelp	-test(Zhelp)	Lagrange (Quadratic)	pt5

1.5.8 Dirichlet Boundary Condition 6

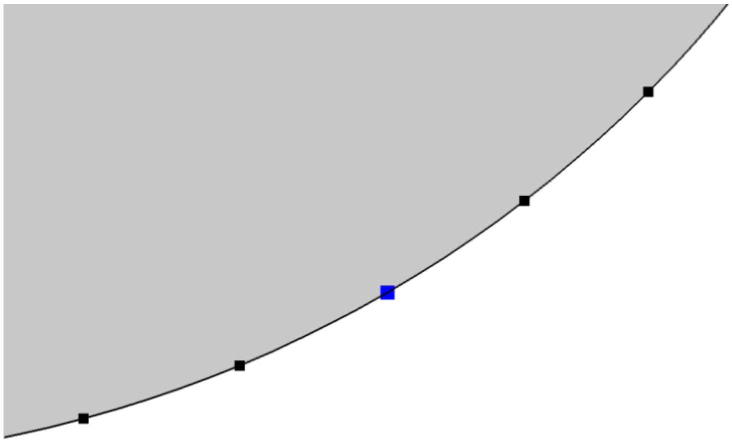

Dirichlet Boundary Condition 6

SELECTION

Geometric entity level	Point
Selection	pt6

EQUATIONS

$$\mathbf{u} = r$$

$\mathbf{u} = [Rhelp, Zhelp]^T$

$g_{reaction} = -\mu$

$\mu = [\mu_1, \mu_2]^T$

SETTINGS

Description	Value
Value on boundary	{Rves*sin(gamma + 4*beta/12) - Rves*sin(4*a), Vp - Rves*cos(gamma + 4*beta/12) + Rves*cos(4*a)}
Prescribed value of Rhelp	On
Prescribed value of Zhelp	On
Apply reaction terms on	Individual dependent variables
Use weak constraints	Off
Constraint method	Elemental

Shape functions

Constraint	Constraint force	Shape function	Selection
Rves*sin(gamma+beta/3)-Rves*sin(4*a)-Rhelp	-test(Rhelp)	Lagrange (Quadratic)	pt6
Vp-Rves*cos(gamma+beta/3)+Rves*cos(4*a)-Zhelp	-test(Zhelp)	Lagrange (Quadratic)	pt6

1.5.9 Dirichlet Boundary Condition 7

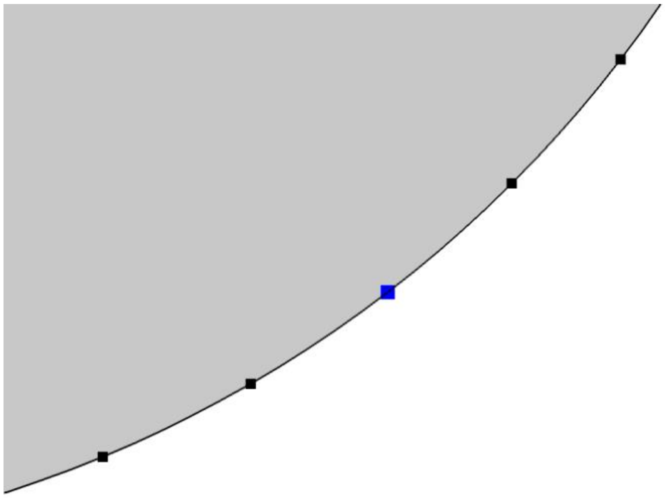

Dirichlet Boundary Condition 7

SELECTION

Geometric entity level	Point

Selection	pt7

EQUATIONS

$$\mathbf{u} = r$$
$$\mathbf{u} = [Rhelp, Zhelp]^T$$
$$g_{\mathrm{reaction}} = -\mu$$
$$\mu = [\mu_1, \mu_2]^T$$

SETTINGS

Description	Value
Value on boundary	{Rves*sin(gamma + 5*beta/12) - Rves*sin(5*a), Vp - Rves*cos(gamma + 5*beta/12) + Rves*cos(5*a)}
Prescribed value of Rhelp	On
Prescribed value of Zhelp	On
Apply reaction terms on	Individual dependent variables
Use weak constraints	Off
Constraint method	Elemental

Shape functions

Constraint	Constraint force	Shape function	Selection
Rves*sin(gamma+0.4166666666666667*beta)-Rves*sin(5*a)-Rhelp	-test(Rhelp)	Lagrange (Quadratic)	pt7
Vp-Rves*cos(gamma+0.4166666666666667*beta)+Rves*cos(5*a)-Zhelp	-test(Zhelp)	Lagrange (Quadratic)	pt7

1.5.10 Dirichlet Boundary Condition 8

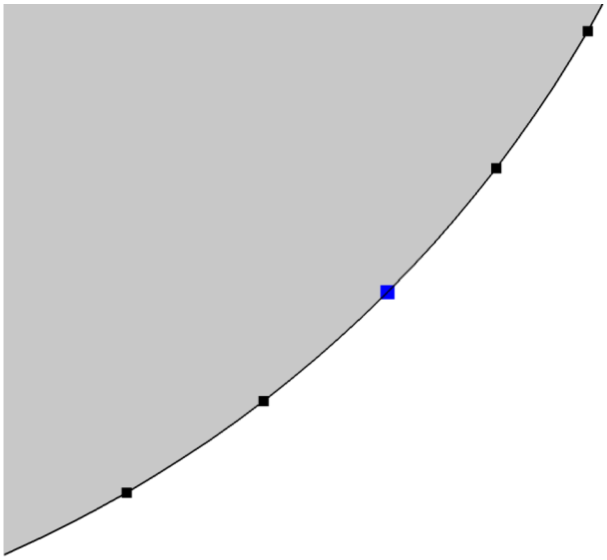

Dirichlet Boundary Condition 8

SELECTION

Geometric entity level	Point
Selection	pt8

EQUATIONS

$$\mathbf{u} = r$$
$$\mathbf{u} = [Rhelp, Zhelp]^T$$
$$g_{reaction} = -\mu$$
$$\mu = [\mu_1, \mu_2]^T$$

SETTINGS

**Description**	**Value**
Value on boundary	{Rves*sin(gamma + 6*beta/12) - Rves*sin(6*a), Vp - Rves*cos(gamma + 6*beta/12) + Rves*cos(6*a)}
Prescribed value of Rhelp	On
Prescribed value of Zhelp	On
Apply reaction terms on	Individual dependent variables
Use weak constraints	Off
Constraint method	Elemental

Shape functions

Constraint	Constraint force	Shape function	Selection
Rves*sin(gamma+0.5*beta)-Rves*sin(6*a)-Rhelp	-test(Rhelp)	Lagrange (Quadratic)	pt8
Vp-Rves*cos(gamma+0.5*beta)+Rves*cos(6*a)-Zhelp	-test(Zhelp)	Lagrange (Quadratic)	pt8

1.5.11 Dirichlet Boundary Condition 9

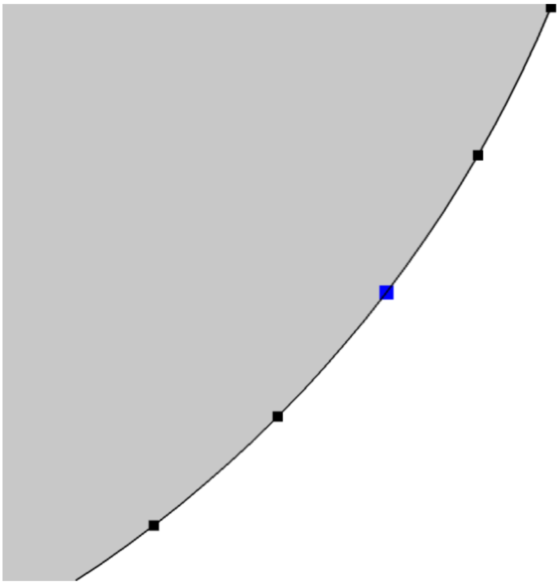

Dirichlet Boundary Condition 9

SELECTION

Geometric entity level	Point
Selection	pt9

EQUATIONS

$$\mathbf{u} = r$$
$$\mathbf{u} = [Rhelp, Zhelp]^T$$
$$g_{\mathrm{reaction}} = -\mu$$
$$\mu = [\mu_1, \mu_2]^T$$

SETTINGS

Description	Value
Value on boundary	{Rves*sin(gamma + 7*beta/12) - Rves*sin(7*a), Vp - Rves*cos(gamma + 7*beta/12) + Rves*cos(7*a)}
Prescribed value of Rhelp	On
Prescribed value of Zhelp	On

Description	Value
Apply reaction terms on	Individual dependent variables
Use weak constraints	Off
Constraint method	Elemental

Shape functions

Constraint	Constraint force	Shape function	Selection
Rves*sin(gamma+0.5833333333333334*beta)-Rves*sin(7*a)-Rhelp	-test(Rhelp)	Lagrange (Quadratic)	pt9
Vp-Rves*cos(gamma+0.5833333333333334*beta)+Rves*cos(7*a)-Zhelp	-test(Zhelp)	Lagrange (Quadratic)	pt9

1.5.12 Dirichlet Boundary Condition 10

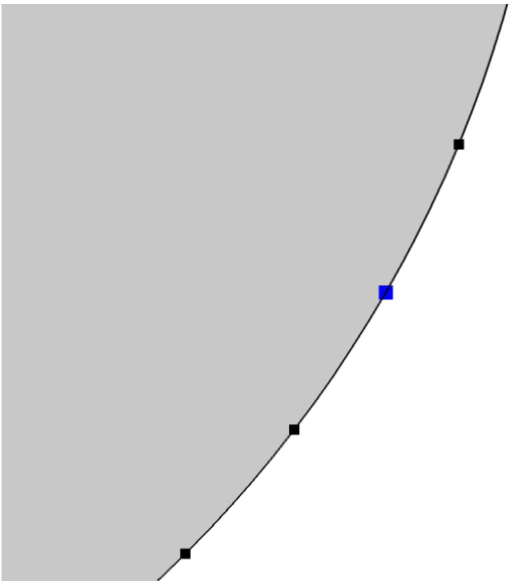

Dirichlet Boundary Condition 10

SELECTION

Geometric entity level	Point
Selection	pt10

EQUATIONS

$$\mathbf{u} = r$$
$$\mathbf{u} = [Rhelp, Zhelp]^T$$
$$g_{\mathrm{reaction}} = -\mu$$
$$\mu = [\mu_1, \mu_2]^T$$

SETTINGS

Description	Value
Value on boundary	{Rves*sin(gamma + 8*beta/12) - Rves*sin(8*a), Vp - Rves*cos(gamma + 8*beta/12) + Rves*cos(8*a)}
Prescribed value of Rhelp	On
Prescribed value of Zhelp	On
Apply reaction terms on	Individual dependent variables
Use weak constraints	Off
Constraint method	Elemental

Shape functions

Constraint	Constraint force	Shape function	Selection
Rves*sin(gamma+2*beta/3)-Rves*sin(8*a)-Rhelp	-test(Rhelp)	Lagrange (Quadratic)	pt10
Vp-Rves*cos(gamma+2*beta/3)+Rves*cos(8*a)-Zhelp	-test(Zhelp)	Lagrange (Quadratic)	pt10

1.5.13 Dirichlet Boundary Condition 11

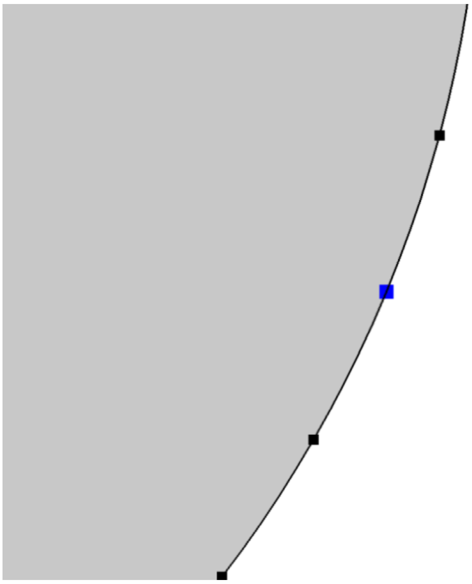

Dirichlet Boundary Condition 11

SELECTION

Geometric entity level	Point
Selection	pt11

EQUATIONS

$\mathbf{u} = r$

$\mathbf{u} = [Rhelp, Zhelp]^T$

$g_{reaction} = -\mu$

$\mu = [\mu_1, \mu_2]^T$

SETTINGS

Description	Value
Value on boundary	{Rves*sin(gamma + 9*beta/12) - Rves*sin(9*a), Vp - Rves*cos(gamma + 9*beta/12) + Rves*cos(9*a)}
Prescribed value of Rhelp	On
Prescribed value of Zhelp	On
Apply reaction terms on	Individual dependent variables
Use weak constraints	Off
Constraint method	Elemental

Shape functions

Constraint	Constraint force	Shape function	Selection
Rves*sin(gamma+0.75*beta)-Rves*sin(9*a)-Rhelp	-test(Rhelp)	Lagrange (Quadratic)	pt11
Vp-Rves*cos(gamma+0.75*beta)+Rves*cos(9*a)-Zhelp	-test(Zhelp)	Lagrange (Quadratic)	pt11

1.5.14 Dirichlet Boundary Condition 12

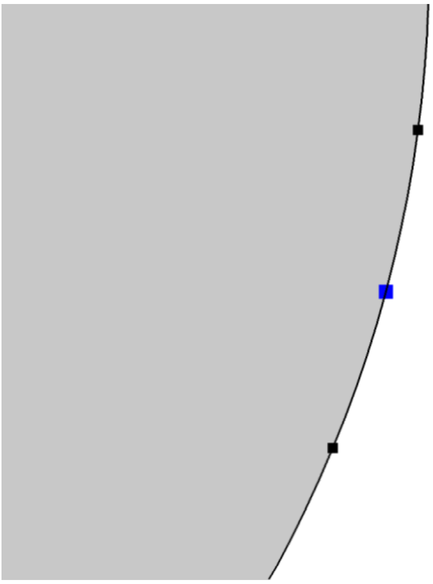

Dirichlet Boundary Condition 12

SELECTION

Geometric entity level	Point
Selection	pt12

EQUATIONS

$$\mathbf{u} = r$$

$$\mathbf{u} = [Rhelp, Zhelp]^T$$

$$g_{\mathrm{reaction}} = -\mu$$

$$\mu = [\mu_1, \mu_2]^T$$

SETTINGS

Description	Value
Value on boundary	{Rves*sin(gamma + 10*beta/12) - Rves*sin(10*a), Vp - Rves*cos(gamma + 10*beta/12) + Rves*cos(10*a)}
Prescribed value of Rhelp	On
Prescribed value of Zhelp	On
Apply reaction terms on	Individual dependent variables
Use weak constraints	Off
Constraint method	Elemental

Shape functions

Constraint	Constraint force	Shape function	Selection
Rves*sin(gamma+0.8333333333333334*beta)-Rves*sin(10*a)-Rhelp	-test(Rhelp)	Lagrange (Quadratic)	pt12
Vp-Rves*cos(gamma+0.8333333333333334*beta)+Rves*cos(10*a)-Zhelp	-test(Zhelp)	Lagrange (Quadratic)	pt12

1.5.15 Dirichlet Boundary Condition 13

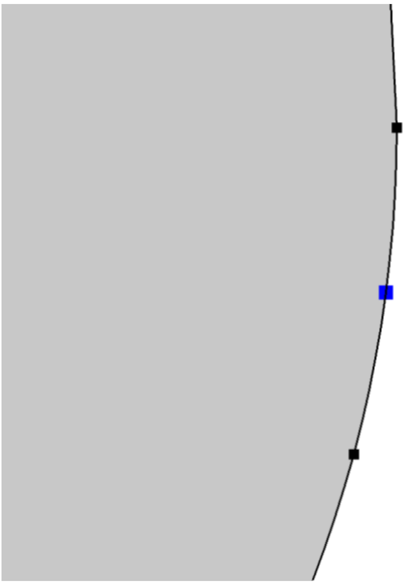

Dirichlet Boundary Condition 13

SELECTION

Geometric entity level	Point
Selection	pt13

EQUATIONS

$$\mathbf{u} = r$$
$$\mathbf{u} = [Rhelp, Zhelp]^T$$
$$g_{\mathrm{reaction}} = -\mu$$
$$\mu = [\mu_1, \mu_2]^T$$

SETTINGS

**Description**	**Value**
Value on boundary	{Rves*sin(gamma + 11*beta/12) - Rves*sin(11*a), Vp - Rves*cos(gamma + 11*beta/12) + Rves*cos(11*a)}
Prescribed value of Rhelp	On
Prescribed value of Zhelp	On
Apply reaction terms on	Individual dependent variables
Use weak constraints	Off
Constraint method	Elemental

Shape functions

Constraint	Constraint force	Shape function	Selection
Rves*sin(gamma+0.9166666666666666*beta)-Rves*sin(11*a)-Rhelp	-test(Rhelp)	Lagrange (Quadratic)	pt13
Vp-Rves*cos(gamma+0.9166666666666666*beta)+Rves*cos(11*a)-Zhelp	-test(Zhelp)	Lagrange (Quadratic)	pt13

1.6 THE TRANSPORT OF DILUTED SPECIES

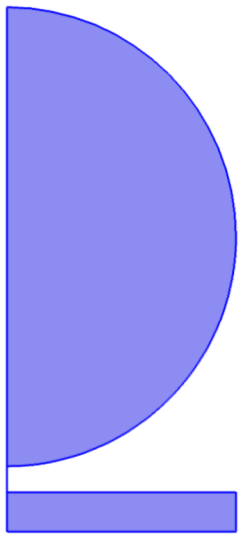

The Transport of Diluted Species

SELECTION

Geometric entity level	Domain
Selection	All domains

EQUATIONS

$$\frac{\partial c_i}{\partial t} + \nabla \cdot (-D_i \nabla c_i) + \mathbf{u} \cdot \nabla c_i = R_i$$

$$\mathbf{N}_i = -D_i \nabla c_i + \mathbf{u} c_i$$

SETTINGS

Description	Value
Concentration	Linear
Compute boundary fluxes	On
Apply smoothing to boundary fluxes	On
Value type when using splitting of complex variables	Real
Convection	On
Migration in electric field	Off
Mass transfer in porous media	Off
Streamline diffusion	On
Crosswind diffusion	On
Equation residual	Approximate residual
Crosswind diffusion type for free flow	Do Carmo and Galeão

Description	Value
Isotropic diffusion	Off
Convective term	Nonconservative form

1.6.1 Transport Properties 1

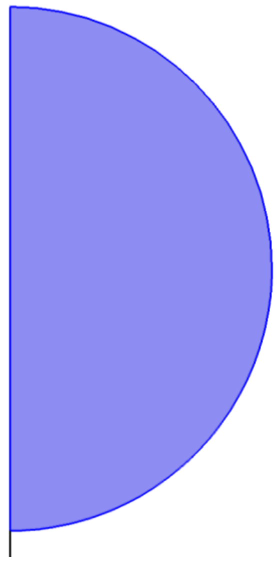

Transport Properties 1

SELECTION

Geometric entity level	Domain
Selection	c1

EQUATIONS

$$\frac{\partial c_i}{\partial t} + \nabla \cdot (-D_i \nabla c_i) + \mathbf{u} \cdot \nabla c_i = R_i$$

$$\mathbf{N}_i = -D_i \nabla c_i + \mathbf{u} c_i$$

SETTINGS

Description	Value
Velocity field	User defined
Velocity field	{0, 0, 0}
Material	None
Diffusion coefficient	User defined
Diffusion coefficient	{{Din, 0, 0}, {0, Din, 0}, {0, 0, Din}}
Temperature	User defined
Temperature	293.15[K]

Shape functions

Name	Shape function	Unit	Description	Shape frame	Selection
c	Lagrange (Linear)	mol/m³	Concentration	Spatial	c1

1.6.2 Initial Values 2

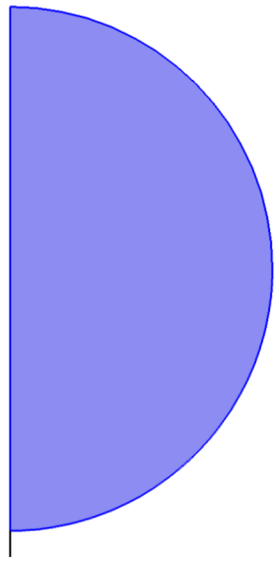

Initial Values 2

SELECTION

Geometric entity level	Domain
Selection	r3, r4

SETTINGS

Description	Value
Concentration	c0

1.6.3 Axis Symmetry 1

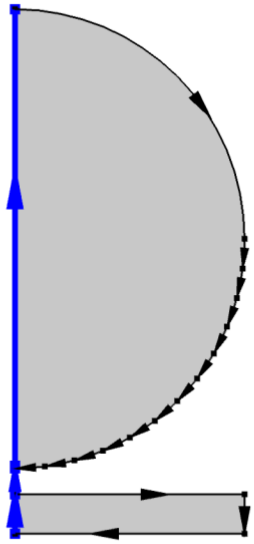

Axis Symmetry 1

SELECTION

Geometric entity level	Boundary
Selection	The symmetric axis

1.6.4 No FLux 1

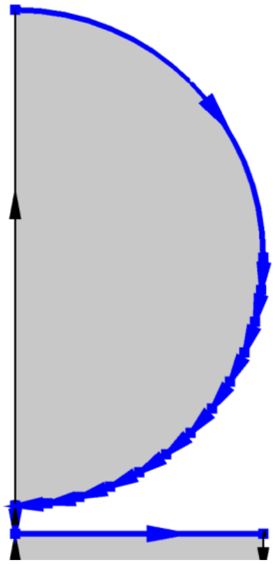

No FLux 1

SELECTION

Geometric entity level	Boundary

Selection	Edges of c1,<br>Right edge of r2,<br>and upper edge of r1

EQUATIONS

$$-\mathbf{n}\cdot\mathbf{N}_i = 0$$

1.6.5 Initial Values 1

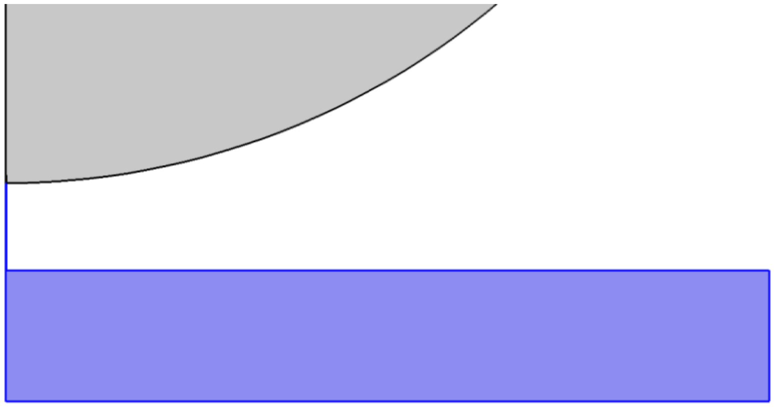

Initial Values 1

SELECTION

Geometric entity level	Domain
Selection	r1, r2

SETTINGS

Description	Value
Concentration	0

1.6.6 Transport Properties 2

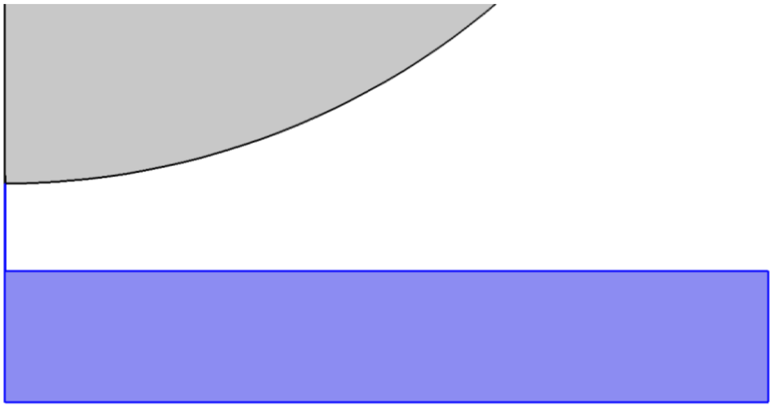

Transport Properties 2

SELECTION

Geometric entity level	Domain
Selection	r1, r2

EQUATIONS

$$\frac{\partial c_i}{\partial t} + \nabla \cdot (-D_i \nabla c_i) + \mathbf{u} \cdot \nabla c_i = R_i$$

$$\mathbf{N}_i = -D_i \nabla c_i + \mathbf{u} c_i$$

SETTINGS

Description	Value
Velocity field	User defined
Velocity field	{0, 0, 0}
Material	None
Diffusion coefficient	User defined
Diffusion coefficient	{{Dout, 0, 0}, {0, Dout, 0}, {0, 0, Dout}}
Temperature	User defined
Temperature	293.15[K]

Shape functions

Name	Shape function	Unit	Description	Shape frame	Selection
c	Lagrange (Linear)	mol/m³	Concentration	Spatial	r1, r2

1.6.7 Concentration 1

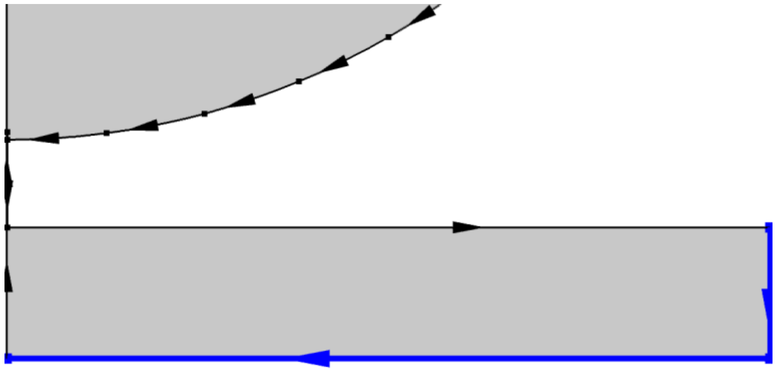

Concentration 1

SELECTION

Geometric entity level	Boundary
Selection	Lower and right edge of r1

EQUATIONS

$$c_i = c_{0,i}$$

SETTINGS

Description	Value
Species c	On
Concentration	0
Apply reaction terms on	All physics (symmetric)
Use weak constraints	Off
Constraint method	Elemental

Variables

Name	Expression	Unit	Description	Selection
tds.c0_c	0	mol/m³	Concentration	Lower and right edge of r1

Shape functions

Constraint	Constraint force	Shape function	Selection
-c+tds.c0_c	test(-c+tds.c0_c)	Lagrange (Linear)	Lower and right edge of r1

1.7 MESHES

1.7.1 Meshes 1

MESH STATISTICS

Description	Value
Minimum element quality	0.5725
Average element quality	0.8843
Triangular elements	1174
Edge elements	438
Vertex elements	22

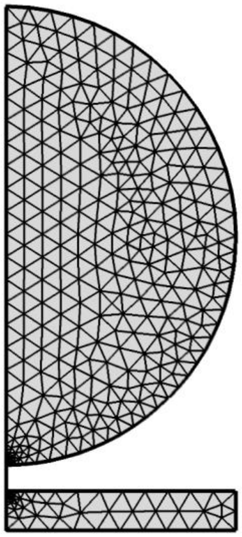

Meshes 1

Size (size)

SETTINGS

Description	Value
Maximum element size	Rves/10
Minimum element size	Rves/200
Curvature factor	0.6
Maximum element growth rate	1.5
Predefined size	Coarser
Custom element size	Custom

Free Triangular MeshesMeshes 1 (ftri1)

SELECTION

Geometric entity level	Domain
Selection	All domains

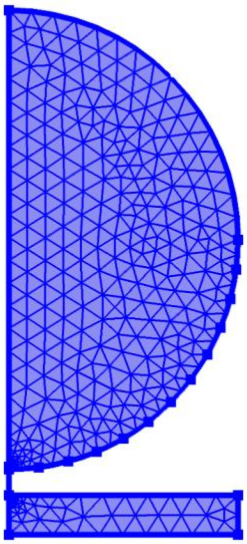

Free Triangular Meshes 1

Size 1 (size1)

SELECTION

Geometric entity level	Point
Selection	2 points on the right edge of r2

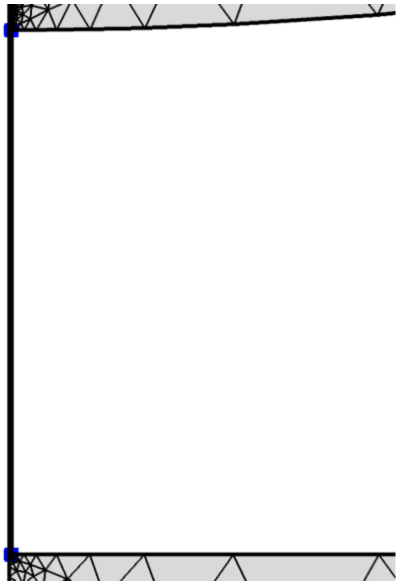

Size 1

SETTINGS

Description	Value
Calibrate for	Semiconductor
Maximum element size	Rves/20
Minimum element size	Rves/200
Curvature factor	0.25
Curvature factor	Off
Resolution of narrow regions	Off
Maximum element growth rate	Off
Predefined size	Finer
Custom element size	Custom

2 Study

COMPUTATION INFORMATION

CPU	Intel(R) Core(TM) i7-8750H CPU @ 2.20GHz, 6 cores
Operating system	Windows 10

Time Dependent	Unit
range(0, 0.1e-3, 20e-3)	s

STUDY SETTINGS

Description	Value
Include geometric nonlinearity	Off

STUDY EXTENSIONS

Description	Value
Automatic remeshing	On

SETTINGS

Description	Value
Remesh in geometry	

PHYSICS AND VARIABLES SELECTION

Physics interface	Discretization
Moving Mesh (ale)	physics
Coefficient Form PDE (cb)	physics
The Transport of Diluted Species (tds)	physics

MESH SELECTION

Geometry	Mesh
Geometry 1	mesh1

REFERENCES

[1] Dunevall, J.; Fathali, H.; Najafinobar, N.; Lovric, J.; Wigstrom, J.; Cans, A. S.; Ewing, A. G., Characterizing the Catecholamine Content of Single Mammalian Vesicles by Collision-Adsorption Events at an Electrode. *J. Am. Chem. Soc.* **2015**, 137, 4344-4346.

[2] Lovric, J.; Najafinobar, N.; Dunevall, J.; Majdi, S.; Svir, I.; Oleinick, A.; Amatore, C.; Ewing, A. G., On the Mechanism of Electrochemical Vesicle Cytometry: Chromaffin Cell Vesicles and Liposomes. *Faraday Discuss.* **2016**, 193, 65-79.

[3] Terejanszky, P.; Makra, I.; Furjes, P.; Gyurcsanyi, R. E., Calibration-Less Sizing and Quantitation of Polymeric Nanoparticles and Viruses with Quartz Nanopipets. *Anal. Chem.* **2014**, 86, 4688-4697.

[4] Weaver, J. C.; Chizmadzhev, Y. A., Theory of Electroporation: A Review. *Bioelectrochemistry and Bioenergetics* **1996**, 41, 135-160.
